# Mapping scientific knowledge on the association between periodontal disease and rheumatoid arthritis: A bibliometric and altmetric analysis of the 100 most-cited articles

**DOI:** 10.4317/medoral.28134

**Published:** 2026-04-09

**Authors:** Lucas Grassi Silvestrini, Maria Fernanda Costa Silva Santos, Bruno de Paula Rodrigues, Laisa Marcelino Gonzaga, Hercílio Martelli Júnior, Gisele Maria Campos Fabri, Marcos Paulo Maia-Lima, Luiz Miguel Ferreira

**Affiliations:** 1 Department of Dental Clinic, School of Dentistry, Federal University of Juiz de Fora, Juiz de Fora, Brazil; 2 Oral Medicine and Oral Pathology, School of Dentistry, State University of Montes Claros, Unimontes, Montes Claros, Brazil; 3 Department of Oral Diagnosis, Piracicaba Dental School, State University of Campinas (FOP-UNICAMP), Piracicaba, São Paulo, Brazil; 4 Department of Dentistry, Estácio de Sá University Center, Juiz de Fora, Brazil

**Keywords:** Rheumatoid arthritis, periodontal diseases, bibliometrics, altmetrics, oral pathology.

## Abstract

**Background:**

A bidirectional relationship between periodontal disease (PD) and rheumatoid arthritis (RA) has been widely investigated, highlighting the potential impact of oral diseases on systemic health. This bibliometric and altmetric analysis aimed to evaluate the 100 most-cited articles exploring the association between PD and RA.

**Materials and Methods:**

A bibliometric review was conducted in accordance with the BIBLIO guidelines. The Scopus database was searched to identify the 100 most-cited articles, and citation counts were cross-validated in the Web of Science database. Data collected included publication year, citation counts, journals, authors, institutions, countries, study design, and collaboration patterns. Bibliometric networks were visualized using VOSviewer, altmetric data were obtained from the Dimensions platform, and public interest trends were analyzed using Google Trends.

**Results:**

The selected articles accumulated 14,753 citations (mean: 147.5 citations per article). Scientific output increased in the 1990s, peaked between 2005 and 2015, and declined thereafter. Research production was concentrated in high-income countries, particularly the United States, the United Kingdom, and Australia, and the resulting articles were published in high-impact journals. The literature primarily focused on molecular mechanisms and biomarkers, whereas clinical trials were comparatively scarce. Altmetric scores demonstrated a weak but statistically significant (p=0.001) positive correlation with citation counts.

**Conclusions:**

This is a well-established, interdisciplinary research field whose academic influence and public relevance are well documented. Nevertheless, limited geographic diversity and a relative paucity of interventional studies remain. Future multicenter and translational investigations are warranted to address these gaps and further advance clinical understanding.

## Introduction

Periodontal disease (PD) is an inflammatory condition affecting the supporting tissues of the teeth. It results from the action of Gram-negative bacteria and a dysregulated host immune response, which leads to the formation of periodontal pockets, destruction of the periodontium, and subsequent bone and tooth loss [1,2]. PD has been extensively investigated because of its impact beyond the oral cavity, particularly in relation to systemic diseases such as rheumatoid arthritis (RA), diabetes mellitus, and pulmonary diseases [3-5]. RA, in turn, is a complex, multifactorial autoimmune inflammatory disorder [6] characterized by chronic joint inflammation [7]. It is frequently initiated in mucosal tissues, such as the lungs, gastrointestinal tract, and oral cavity, and is associated with alterations in the microbiota [2,8].

Evidence suggests that periodontal inflammation may act as a risk factor for chronic systemic conditions, including RA [1], through immunoinflammatory mechanisms and microbial dysbiosis [2,9]. A bidirectional relationship between PD and RA has been frequently reported in the scientific literature [4], suggesting that periodontal inflammation may not only exacerbate RA progression but may also be influenced by the systemic inflammatory state associated with this autoimmune disorder [10].

The term bibliometrics was first introduced in 1969 by Professor Alan Pritchard, who defined it as the application of mathematical and statistical methods to books and other media of communication [11]. Since then, bibliometric analysis has emerged as a valuable tool for quantifying and mapping trends in scientific literature, enabling the identification of influential publications, leading authors and institutions, as well as research gaps that warrant further investigation [3,12].

Furthermore, with the increasing adoption of alternative metrics, such as altmetrics, it has become possible to assess not only the academic relevance of publications but also their dissemination and societal impact, thereby complementing traditional indicators such as citation counts and journal impact factors [12]. Conventional metrics typically require years to consolidate after publication. In contrast, altmetric data can be obtained rapidly, with updates occurring in real time or on a daily basis across platforms such as Wikipedia, Google, X, and Facebook [13].

Relevant topics in oral pathology have been extensively investigated through bibliometric studies [14]. However, to date, no bibliometric studies have analyzed the publication patterns and metrics related to the association between PD and RA. Therefore, a bibliometric review was conducted to evaluate and quantify the 100 most-cited articles addressing PD in patients with RA. The aim was to map the knowledge structure of the scientific literature in this field; identify emerging themes, leading researchers, and influential journals; and delineate their interrelationships, thereby providing comprehensive insights into this research domain.

## Materials and Methods

*Design, information sources and search strategy*

A bibliometric review was conducted in accordance with the BIBLIO guidelines for reporting bibliometric reviews of the biomedical literature [15]. On August 25, 2025, a search was performed in the Scopus database, selected for its broad journal coverage, using a quantitative approach [16]. Articles were identified using the following search strategy: [("arthritis, rheumatoid" OR "rheumatoid arthritis") AND ("disease, periodontal" OR "diseases, periodontal" OR "periodontal disease" OR "periodontitis" OR "periodontal atrophy" OR "tooth loss" OR "parodontosis" OR "parodontoses" OR "periodontal pocket" OR "alveolar bone loss" OR "periodontal bone loss" OR "gingival recession" OR "pyorrhea alveolaris" OR "periodontal attachment loss" OR "periodontal clinical attachment loss" OR "aggressive periodontitis" OR "chronic periodontitis")]. 

*Eligibility criteria*

In this bibliometric analysis, studies addressing PD in patients with RA were included, with no restrictions on publication year or language. Publications not directly related to the topic, as well as studies that provided only a superficial discussion or that investigated the association between PD and other rheumatologic conditions, were excluded. Studies for which full-text access was unavailable were also excluded.

*Selection process and data cross-referencing*

The retrieved records were organized according to academic relevance, as measured by citation counts. The top 100 most-cited studies were selected for analysis by two independent reviewers (L.G.S. and L.M.F.), who screened the titles and abstracts and, when necessary, assessed the full texts. Any disagreements were resolved through discussion until consensus was reached. When consensus could not be achieved, a third researcher (M.P.M.L.) was consulted. To enhance reliability, citation counts of the selected articles were cross-validated with records from the Web of Science (WoS) database. This strategy was adopted due to variations in indexing criteria and database coverage across platforms, which may substantially influence the scope of bibliometric analyses. In cases of tied rankings, priority was given to the article published earlier.

*Data extraction*

From the selected articles, the following information was extracted: Title, language, number and names of authors, citation counts in Scopus and WoS, institution, country, and continent (based on the corresponding author’s affiliation), year of publication, journal, keywords, study design, and study topic. Study design was categorized as follows: Review, laboratory study, observational study, and interventional study (clinical trial). Study topics were grouped into the following categories: Epidemiology and clinical associations; reviews and general concepts; experimental models and animal studies; treatment and therapeutic impact; and molecular mechanisms and biomarkers.

Additionally, the following data were collected: 2024 impact factor (IF), publishers’ names, and the country of origin of the main journals listed in the Journal Citation Reports (JCR). Journal quartile classification and h-index were retrieved from the SCImago Journal & Country Rank (SJR) database for the year 2024. To establish the profiles of leading researchers, their individual records were accessed directly in the Scopus and WoS databases to obtain their total number of publications, total citations, and h-index.

*Bibliometric and altmetric analysis*

Microsoft® Excel® (Version 2508, Build 16.0.19127.20192) was used to generate the graph illustrating the distribution of articles and citations over time. The Visualization of Similarities Viewer (VOSviewer, version 1.6.17) was employed to produce graphical representations of bibliometric networks, highlighting relationships among authors and keywords. The MapChart platform (https://www.mapchart.net/) was used to construct a world map depicting the country-level distribution of the selected articles. Altmetric data were retrieved from the Dimensions platform (https://www.dimensions.ai/). Additionally, the global popularity of the search terms “periodontal disease” and “rheumatoid arthritis” was assessed using Google Trends (https://trends.google.com/trends/).

*Statistical analysis*

Data were organized in Microsoft Excel, and statistical analyses were performed using SPSS® (Statistical Package for the Social Sciences, version 27.0; IBM Corp., Armonk, NY, USA). The Kolmogorov-Smirnov test was applied to assess data normality. Spearman’s rank correlation test was used to analyze associations among bibliometric metrics, year of publication, and number of authors. The Kruskal-Wallis test was employed to compare citation counts across publication decades, corresponding author’s continent, study design, and study topic. For the dichotomous variable international collaboration, the Mann-Whitney U test was applied. A significance level of 5% was adopted (*p*≤0.05).

## Results

Following the predefined search strategy, 2,264 documents were initially identified. To obtain the final sample of the 100 most-cited articles, records were screened in descending order of citation count. A total of 417 papers were evaluated; of these, 317 were excluded for not meeting the inclusion criteria, resulting in a final set of the 100 most-cited articles on PD and RA Supplementary 1 (https://www.medicinaoral.com/medoralfree01/28134/Suppl1.pdf).

*Citation analysis*

The 100 selected articles received a total of 14,753 citations in Scopus, with a mean of 147.53 per article (SD=97.92). Citation counts ranged from 67 to 577, with 19% of the articles receiving more than 200 citations, including 10% that received at least 300. In the WoS database, 12,852 citations were recorded, with a mean of 128.52 per article (SD=86.89), ranging from 0 to 494 (https://www.medicinaoral.com/medoralfree01/28134/Suppl1.pdf). A strong positive correlation was observed between citation counts in Scopus and WoS (rho=0.976; *p<*0.001), indicating that articles highly cited in one database tended to be similarly cited in the other.

The most-cited article in both databases was “Peptidylarginine deiminase from Porphyromonas gingivalis citrullinates human fibrinogen and α-enolase: Implications for autoimmunity in rheumatoid arthritis,” a laboratory study conducted by Wegner *et al*. [17], published in 2010 in Arthritis & Rheumatism (United Kingdom). This article had an average of 38.47 citations per year in Scopus Supplementary 1 (https://www.medicinaoral.com/medoralfree01/28134/Suppl1.pdf).

Statistical analysis https://www.medicinaoral.com/medoralfree01/28134/Suppl1.pdf indicated that, although laboratory studies and review articles addressing the topics “reviews and general concepts” and “epidemiology and clinical associations,” as well as articles involving international collaboration, tended to receive higher citation counts, these differences were not statistically significant. These findings suggest that, within this set of 100 articles, these factors did not exert a consistent influence on citation impact.

*Year of publication*

The oldest article, “Serum antibodies and loss of periodontal bone in patients with rheumatoid arthritis,” was published in 1990 by Tolo and Jorkjend and has received 81 citations in Scopus. The most recent articles were published in 2021: “Periodontitis and rheumatoid arthritis: What have we learned about their connection and their treatment?” by González-Febles and Sanz (93 citations in Scopus), and “Dysbiosis in the oral microbiomes of anti-CCP-positive individuals at risk of developing rheumatoid arthritis” by Cheng *et al*. (67 citations in Scopus) Supplementary 1 (https://www.medicinaoral.com/medoralfree01/28134/Suppl1.pdf).

The temporal analysis of publications and citations revealed three phases: Gradual growth (1990-2005), peak productivity and scientific impact (2005-2015), and a recent decline (2015-2025), possibly reflecting the maturation of the field or a shift toward more specialized subtopics (Figure 1).

Spearman’s correlation indicated a weak positive association between publication year and the number of authors (rho=0.303; *p=*0.002), suggesting that more recent articles tend to have a higher number of authors. Conversely, publication year exhibited a weak negative correlation with citation counts in Scopus (rho=-0.373; *p<*0.001) and in the Web of Science (rho=-0.335; *p=*0.001), indicating that older articles tend to accumulate more citations across these databases.

Significant differences were also observed in citation counts by decade (Scopus: *p=*0.016; WoS: *p=*0.037). Articles published in the 2000s had the highest mean number of citations, followed by those from the 2010s and 1990s, whereas the most recent publications (2020s) exhibited the lowest impact, reflecting the time required for academic consolidation https://www.medicinaoral.com/medoralfree01/28134/Suppl1.pdf

*Countries and continents*

The United States led in the number of publications, contributing 24% of the articles, followed by the United Kingdom (12%), Australia (11%), Brazil (7%), Germany (6%), and Japan, Turkey, and Sweden (5% each). France and the Netherlands completed the top 10 with 4% and 3%, respectively. The citation impact analysis, measured by citation counts in Scopus, revealed that the United States again ranked highest (4,427; 30%), followed by the United Kingdom (2,256; 15.3%) and Australia (1,854; 12.6%).

Statistical analysis Supplementary 1 (https://www.medicinaoral.com/medoralfree01/28134/Suppl1.pdf) revealed a significant difference in citation counts among continents in the Scopus database (*p=*0.043), with articles from the Americas and Oceania receiving, on average, more citations than those from Europe and Asia. No significant difference was observed in the Web of Science database (*p=*0.065). The geographic distribution (Figure 2) demonstrated a concentration of research in developed countries, particularly in the Northern Hemisphere and Oceania, with no studies originating from Africa, highlighting a substantial geographic gap in scientific output on this topic.

*Contributing journals*

The journals with the highest representation among the 100 most-cited articles were led by the Journal of Periodontology (14 articles; 2,215 citations), followed by the Journal of Clinical Periodontology (12 articles; 1,589 citations; Table 1). Most of these journals were based in countries with high scientific output, such as the United States and the United Kingdom, and published by major publishers, including Wiley, Elsevier, and Nature. Several also possessed a high impact factor, such as Nature Reviews Rheumatology (IF 32.7) and Annals of the Rheumatic Diseases (IF 20.6). All were classified in the first quartile (Q1), placing them among the top 25% of journals in terms of relevance and prestige within the field. Furthermore, these journals combine high productivity with academic influence, as reflected in their elevated H-index values, including 363 for Arthritis & Rheumatology and 300 for Annals of the Rheumatic Diseases. These findings indicate that frequent publications in these journals are associated with greater scientific impact and recognition.

*Contributing institutions*

A total of 389 institutions were affiliated with the studies included in this review. Table 2 presents the ten institutions with the highest number of publications, with the University of Adelaide (1,142 citations in Scopus; 7.7%), the Kennedy Institute of Rheumatology (1,142 citations in Scopus; 7.7%), and the University of Nebraska (848 citations in Scopus; 5.8%) emerging as the three institutions with the highest citation counts.

*Contributing authors*

A total of 548 authors were identified across the 100 selected articles. The most productive author was Bartold PM (Australia), with 11 articles and 1,832 citations, followed by Potempa J (Poland) with 8 articles and 1,822 citations, and Venables PJ (United Kingdom) with 7 articles and 1,492 citations. These researchers demonstrate extensive indexed output in databases such as WoS and Scopus, reflecting not only the number of highly cited articles but also their overall productivity and academic impact. For instance, Bartold PM exhibits high h-index values (75 in WoS and 70 in Scopus), indicating consistent scientific influence. Mikuls TR and Dietrich T, despite having fewer articles among the 100 most-cited, show a high total number of citations and substantial h-index values, highlighting their significant contributions to scientific research. Furthermore, the geographic distribution indicates that the leading authors are primarily located in developed countries with strong traditions in biomedical research, including Australia, the United States, the United Kingdom, Poland, and Sweden, as shown in Table 3.

The bibliometric network analysis (Figure 3) revealed a complex collaborative structure organized into three thematic clusters, with Potempa J emerging as the most central author and a key link between different communities. These clusters indicate specialized subfields in which researchers with thematic or methodological affinities collaborate more intensively. The density and diversity of the connections highlight a mature and interconnected field, characterized by local collaborations within clusters and strategic bridges that facilitate knowledge transfer across specialties, underscoring the interdisciplinary and collaborative nature of the area.

*Study design and topics*

In terms of study design, 42 observational studies were identified, accounting for 5,754 citations in Scopus (39% of the total citations), followed by 30 review articles (4,762 citations; 32.3%), 19 laboratory studies (3,096 citations; 21%), and 9 clinical trials (1,141 citations; 7.7%) Supplementary 1 (https://www.medicinaoral.com/medoralfree01/28134/Suppl1.pdf).

Regarding research topics, “molecular mechanisms and biomarkers” was the most frequent (n=43), accounting for 6,553 citations in Scopus (44.4%). The second most common field was “epidemiology and clinical associations” (n= 25), with 3,833 citations (26%). The categories “reviews and general concepts” and “treatment and therapeutic impact” each comprised 11 articles, which accrued 1,840 citations (12.5%) and 1,386 citations (9.4%), respectively. The least explored topic was “experimental models and animal studies,” represented by 10 articles with 1,141 citations (7.7%) Supplementary 1 (https://www.medicinaoral.com/medoralfree01/28134/Suppl1.pdf).

*Key words*

Regarding the most frequently occurring keywords among the 100 selected articles, a total of 1,124 distinct terms were identified. “Rheumatoid arthritis” was the most prominent (94 occurrences), followed by “human” (87 occurrences), “periodontitis” (81 occurrences), “male” (61 occurrences), “female” (60 occurrences), and Porphyromonas gingivalis (55 occurrences), as shown in Figure 4.

*Altmetric analysis*

The laboratory study “Aggregatibacter actinomycetemcomitans-induced hypercitrullination links periodontal infection to autoimmunity in rheumatoid arthritis” by Konig *et al*. [18] ranked first, with extensive dissemination across social and scientific media, achieving an altmetric score of 463. Maresz *et al*. and Kaur, White, and Bartold also received high altmetric scores (130 and 52, respectively) (Figure 5). Only the top ten articles were included, as the altmetric values for the remaining publications were low. Notably, laboratory studies and systematic reviews demonstrated that both experimental investigations and evidence syntheses attracted considerable online engagement and media coverage. Moreover, although Wegner *et al*. [17] recorded the highest number of citations in Scopus and WoS, it did not achieve the highest altmetric score. The article with the second-highest citation count, Konig *et al*. [18], ranked first in altmetric performance.

The altmetric score showed a significant but weak positive correlation with citations in Scopus (rho=0.332; *p=*0.001) and in Web of Science (rho=0.319; *p=*0.001), indicating that articles with higher traditional citation counts also tend to attract greater attention in alternative metrics. No significant correlations were found between the altmetric score and the year of publication (rho=0.013; *p=*0.899) or the number of authors (rho=0.137; *p=*0.173).

*Google Trends analysis*

Based on Google Trends data Supplementary 1 (https://www.medicinaoral.com/medoralfree01/28134/Suppl1.pdf) between June 2004 and September 2025, “periodontal disease” showed an average interest of 35%, which was lower than that observed for “rheumatoid arthritis” (66%). In addition, the number of searches for RA was higher than that for PD during the majority of the study period, with only one period of sharp decline between April and June 2020. Japan had the highest search volume for “periodontal disease,” followed by Taiwan, Hong Kong, the United Kingdom, and Ireland. The most common related search topics included medical treatment and the International Statistical Classification of Diseases and Related Health Problems, 10th Revision (ICD-10). For “rheumatoid arthritis,” the countries with the highest interest were Puerto Rico, Egypt, the United States, Norway, and Bolivia, with the most frequent related topics including signs and symptoms as well as diagnosis.

## Discussion

To the best of our knowledge, we are the first to conduct a combined bibliometric and altmetric analysis addressing the relationship between RA and PD. This analysis maps historical trends, influential research centers, high-impact journals, predominant research themes, and their broader societal impact [3,12]. The findings indicate the consolidation of an interdisciplinary field while also highlighting persistent challenges that remain to be addressed.

The citation analysis revealed high academic visibility, with 14,753 citations in Scopus and a strong correlation with WoS records, indicating consistent patterns of citation impact. The most-cited article, by Wegner *et al*. [17], described the citrullination of human proteins by peptidylarginine deiminase produced by Porphyromonas gingivalis, suggesting a possible mechanistic link between PD and autoimmunity in RA. Other influential studies by Konig *et al*. [18] and Scher *et al*. [6] further explored this relationship through the role of Aggregatibacter actinomycetemcomitans and alterations in the oral microbiome of RA patients, illustrating the progression from epidemiological associations to molecular mechanisms.

Analyses of publications in dentistry [19] have demonstrated shifts in study profiles and thematic areas as scientific research and technologies evolve [20], influencing the topics examined in bibliometric investigations. Despite these advances, the causal relationship between PD and RA remains debated [21], as much of the available evidence derives from experimental models or observational studies that may be subject to confounding bias. Although scientific output is traditionally evaluated using bibliometric indicators such as citation counts and journal impact factors [3,22,23], these metrics do not fully capture research influence beyond academia, particularly concerning societal impact and clinical practice [23].

The temporal analysis identified three phases: Gradual growth between 1990 and 2005, peak productivity between 2005 and 2015, and a subsequent relative decline. Similar patterns have been observed in other studies, in which productivity has remained stable or decreased in recent years [24,25]. This reduction may reflect the maturation of the field and a redirection of research toward more specific subtopics. The lower citation counts observed in more recent articles likely reflect the time required for citations to accumulate and for academic impact to consolidate [26].

Geographically, scientific output is concentrated in developed countries, with the United States, the United Kingdom, and Australia accounting for nearly half of all publications, consistent with findings from other dental bibliometric studies [19]. This predominance likely reflects established research infrastructure and greater funding availability. However, the absence of contributions from African countries and the underrepresentation of Latin American research highlight persistent global disparities in scientific production. Such imbalances may affect the generalizability of findings, particularly given that genetic, environmental, and socioeconomic factors may influence both PD and RA. Higher citation rates from the Americas and Oceania suggest greater international recognition [27], possibly reflecting linguistic and editorial biases favoring English-language publications.

The University of Adelaide, the Kennedy Institute of Rheumatology, and the University of Nebraska emerged as leading research centers. Bartold PM, Potempa J, and Venables PJ were the most prominent authors in terms of publications and citations. Co-authorship network analysis revealed collaborative research lines integrating microbiology, immunology, and clinical practice. The presence of bridge authors connecting different clusters suggests a mature and well-integrated research field.

Journal analysis reinforces the importance of high-impact publications in consolidating this research topic. The Journal of Periodontology and the Journal of Clinical Periodontology, as previously reported by Aljabbary, Ul Haq, and Shujaat [28], led in the number of publications, demonstrating their central role as primary outlets for periodontology research. Prestigious multidisciplinary journals, such as Nature Reviews Rheumatology and Annals of the Rheumatic Diseases, contributed to expanding the visibility of this topic by reaching audiences beyond dentistry, reflecting the interdisciplinary nature of RA research [29]. The predominance of Q1 journals with high H-index values further confirms that this topic is disseminated through highly selective scientific channels.

Observational studies (42%) and review articles (30%) were the most prevalent study designs and accounted for the majority of citations. However, clinical trials were relatively scarce (only 9%), which limits the translation of current knowledge into therapeutic recommendations. The bibliometric findings suggest an active research field; nevertheless, whether periodontal treatment reduces inflammatory markers or attenuates RA activity remains to be confirmed through well-designed clinical trials [30]. Nearly half of the articles (44.4%) focused on molecular mechanisms and biomarkers, reflecting a strong emphasis on the interface between microbiology, immunology, and rheumatology. This concentration indicates ongoing efforts to elucidate the biological mechanisms underlying the PD-RA relationship beyond simple epidemiological correlations.

The keyword analysis revealed a structured thematic organization, with high-frequency descriptors concentrated in dense areas of the map, indicating widely addressed and interconnected topics [31]. Overlapping clusters suggest that highly cited studies tend to integrate multiple conceptual approaches. Demographic, laboratory, and methodological terms structure much of the literature, whereas less frequent keywords appear in peripheral regions, representing emerging or underexplored subtopics [32]. The density map therefore highlights both consolidated research cores and potential knowledge gaps, reflecting a well-structured yet evolving research field.

Recent studies have emphasized the importance of altmetric indicators for more comprehensively assessing the dissemination and broader impact of scientific research [33,34]. Social interactions and digital visibility surrounding scientific articles may influence their adoption in clinical practice and public policy discussions [12,19]. Recent analyses indicate that dentistry has undergone notable changes in the way research findings are disseminated and discussed in digital environments [35,36]. Unlike academic citations, which accumulate gradually over time, altmetric indicators capture real-time engagement from both scientific and non-scientific audiences. These indicators include mentions on social media platforms, news outlets, blogs, Wikipedia, clinical guidelines, and patents, thereby reflecting the broader reach and societal impact of research outputs [12].

The altmetric analysis revealed that several articles with high scientific impact also achieved substantial digital visibility. The article by Konig *et al*. [18], published in Science Translational Medicine, received the highest Altmetric Attention Score, with extensive coverage in news outlets (51 items), social media (more than 80 mentions on X/Twitter), and Wikipedia pages. This pattern illustrates how findings linking PD to key mechanisms of autoimmunity, such as leukotoxin-induced hypercitrullination associated with Aggregatibacter actinomycetemcomitans, can extend beyond academic interest and reach broader audiences. It also reflects how the relationship between oral health and systemic diseases attracts public and media attention, particularly when findings highlight the potential impact of oral conditions on systemic diseases such as RA [37].

Furthermore, articles with strong translational appeal, particularly those published in prestigious multidisciplinary journals, appear more likely to gain visibility in digital environments, thereby expanding the societal reach of dentistry-related research [38,39]. However, unlike traditional citations, altmetric indicators can be volatile and are strongly influenced by contextual factors [39]. They do not necessarily reflect methodological quality or scientific rigor and may, in some cases, amplify studies that attract media attention despite limited evidence. Consequently, altmetrics should be interpreted as complementary indicators alongside traditional bibliometric measures and qualitative evaluations of scientific content.

The data also reveal a consistent pattern of greater global search interest in “rheumatoid arthritis” compared with “periodontal disease” over the past two decades. Although both terms show relative stability over time, RA exhibits more pronounced peaks and higher average levels of search interest, reflecting greater public visibility and perceived clinical relevance [38,39]. The decline observed between April and June 2020 likely reflects changes in public search behavior during the early stages of the COVID-19 pandemic.

Geographically, PD shows higher search interest in several Asian countries and in the United Kingdom, whereas RA demonstrates greater predominance in the Americas, the Middle East, and Europe. Related topics also differ in terms of public engagement. PD is frequently associated with diagnostic and therapeutic classifications, whereas RA attracts attention mainly related to symptoms and clinical diagnosis [40]. These findings suggest that, despite the high prevalence of PD, public awareness of the condition appears to be more geographically concentrated compared with that of systemic conditions such as RA.

Several limitations should be acknowledged. Bibliometric analyses depend on database coverage, and indexing criteria may introduce bias. Although Scopus data were cross-validated with WoS records, reliance on a single primary database may still influence citation rankings. Citation counts do not necessarily reflect methodological quality and may be affected by language, disciplinary differences, journal visibility, and self-citation practices. The “Top 100 most-cited” study design also favors older publications, potentially underrepresenting more recent studies. In addition, Google Trends analyses are sensitive to term selection, language, geographic region, and temporal context, and should therefore be interpreted as complementary indicators of public interest. Altmetric data were available mainly for highly visible articles, which may limit broader generalization. Nevertheless, the combined bibliometric, altmetric, and collaboration network analyses provide a comprehensive overview of the scientific landscape of this research field.

## Conclusions

Research on the association between PD and RA has received sustained scientific attention, as reflected in citation patterns, interdisciplinary collaborations, and publications in high-impact journals. Bibliometric and altmetric indicators demonstrate substantial academic and societal visibility but do not directly assess methodological quality. The predominance of observational and experimental studies, combined with a limited number of interventional investigations, characterizes the current state of the field. Future multicenter and translational studies, particularly in underrepresented regions, are needed to strengthen the evidence base.

## Supplementary Material

Supplementary material 1

Supplementary material 2

Supplementary material 3

Supplementary material 4

## Figures and Tables

**Table 1:** Table 1: The 10 most frequently cited journals among the 100 most-cited articles on the relationship between periodontal disease and rheumatoid arthritis.

**Source title**	**Number of papers**	**Number of citations (Scopus)**	**Country**	**Publisher**	**Impact factor (2024)**	**Quartile (2024)**	*h* **-index**
Journal of Periodontology	14	2,215	United States	Wiley	3.8	Q1	186
Journal of Clinical Periodontology	12	1,589	Denmark	Wiley	6.8	Q1	187
Annals of the Rheumatic Diseases	8	863	United Kingdom	Elsevier	20.6	Q1	300
Arthritis Research and Therapy	5	680	United Kingdom	BMC	4.6	Q1	184
Arthritis and Rheumatism	4	1,353	United States	Wiley-Blackwell	-	-	-
Nature Reviews Rheumatology	3	1,044	United States	Nature Portfolio	32.7	Q1	198
Journal of Rheumatology	3	635	Canada	J Rheumatol Publ Co	3.4	Q1	201
Arthritis and Rheumatology	3	556	United States	Wiley	10.9	Q1	363
Current Opinion in Rheumatology	3	289	United States	Lippincott Williams and Wilkins	4.3	Q1	132
Scientific Reports	2	274	United Kingdom	Nature portfolio	3.9	Q1	347

**Table 2:** Table 2: Institutions most frequently represented among the 100 most-cited articles on the relationship between periodontal disease and rheumatoid arthritis.

**Institution**	**Country**	**Number of papers**	**Number of citations (Scopus)**
University of Adelaide	Australia	8	1142
Niigata University	Japan	5	529
Kennedy Institute of Rheumatology	United Kingdom	4	1142
University of Nebraska	United States	4	848
Johns Hopkins University	United States	4	787
Karolinska Institutet	Sweden	3	288
University of Sao Paulo	Brazil	3	280
University of Leeds	United Kingdom	3	264
University of Queensland	Australia	2	605
Charité - Universitätsmedizin Berlin	Germany	2	498

**Table 3:** Table 3: The ten authors with the highest number of articles among the 100 most-cited articles on the relationship between periodontal disease and rheumatoid arthritis.

**Authors**	**Country**	**Number of papers among the 100 most-cited**	**Number of citations among 100 most-cited (Scopus)**	**Number of papers in WoS**	**Number of papers in Scopus**	**Number of citations in WoS**	**Number of citations in Scopus**	*h* **-index in WoS**	*h* **-index in Scopus**
Bartold PM	Australia	11	1,832	502	347	18,450	18,640	75	70
Potempa J	Poland	8	1,822	381	489	8,535	25,473	58	82
Venables PJ	United Kingdom	7	1,492	246	155	7,326	10,416	52	53
Mikuls TR	United States	6	1,242	693	370	12,546	20,404	62	72
Lundberg K	Sweden	5	1,161	119	79	3,339	7,834	35	38
Marshall RI	Australia	4	1,024	27	27	1,363	2,118	16	19
Dietrich T	United Kingdom	4	916	130	184	12,548	20,242	50	55
De Pablo P	United Kingdom	4	916	98	52	2,474	3,255	24	27
Payne JB	United States	4	849	54	71	1,935	3,682	27	30
Thiele GM	United States	4	849	498	248	5,953	8,650	47	49

WoS: Web of Science.

**Figure 1:**
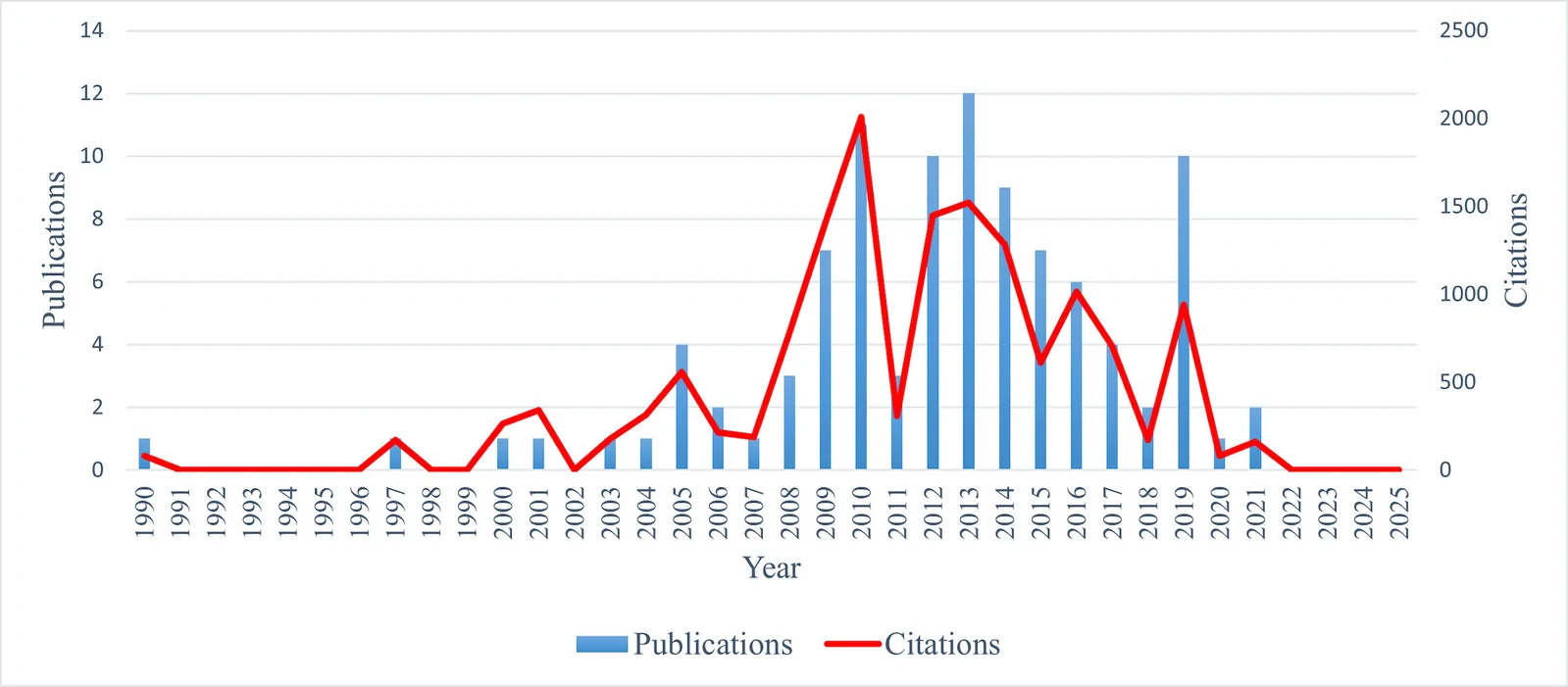
Fig. 1: Distribution of the number of publications and citations over the years in the Scopus database.

**Figure 2:**
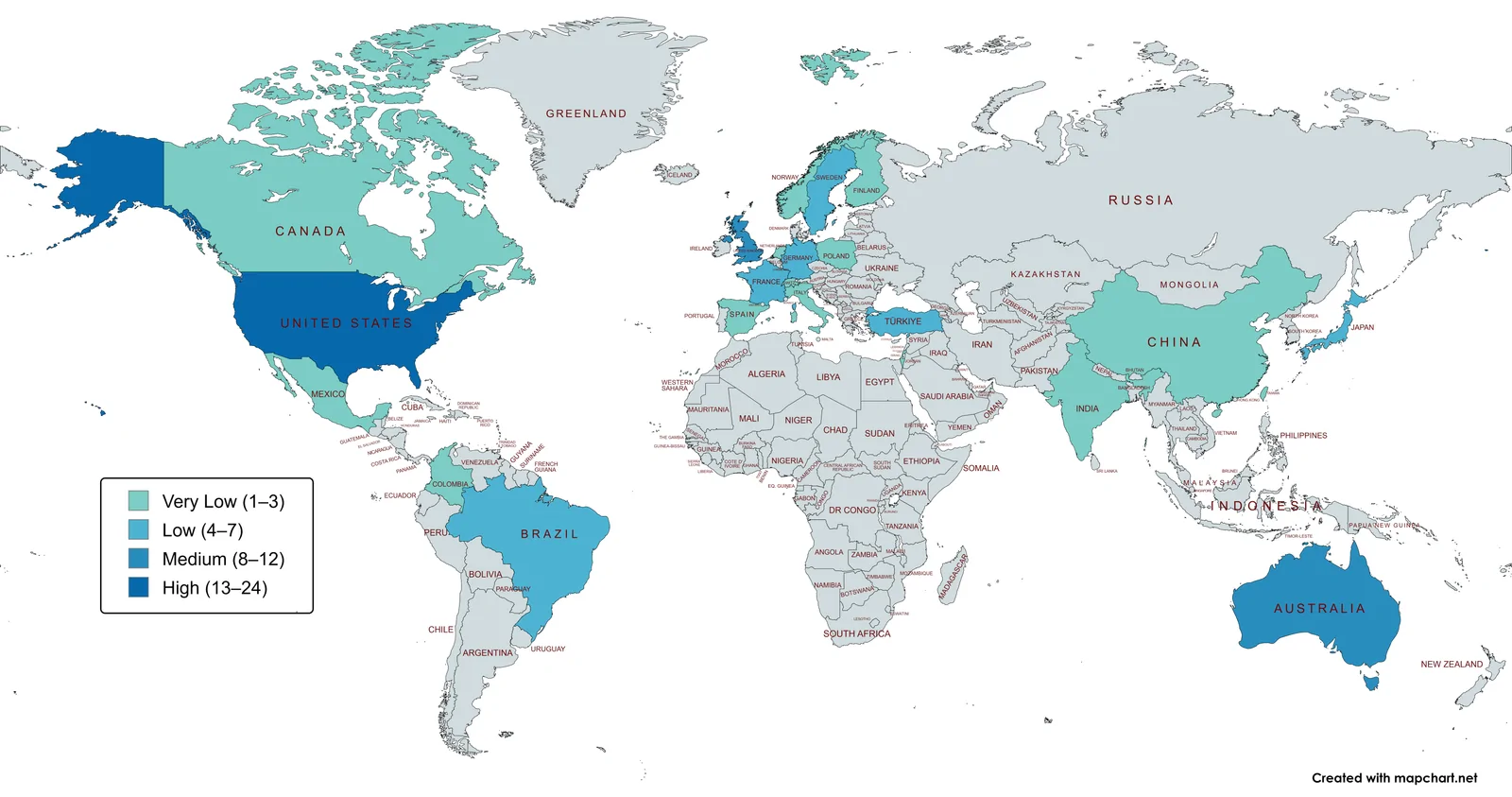
Fig. 2: Geographical distribution of countries with publications on periodontal disease and rheumatoid arthritis among the 100 most-cited articles.

**Figure 3:**
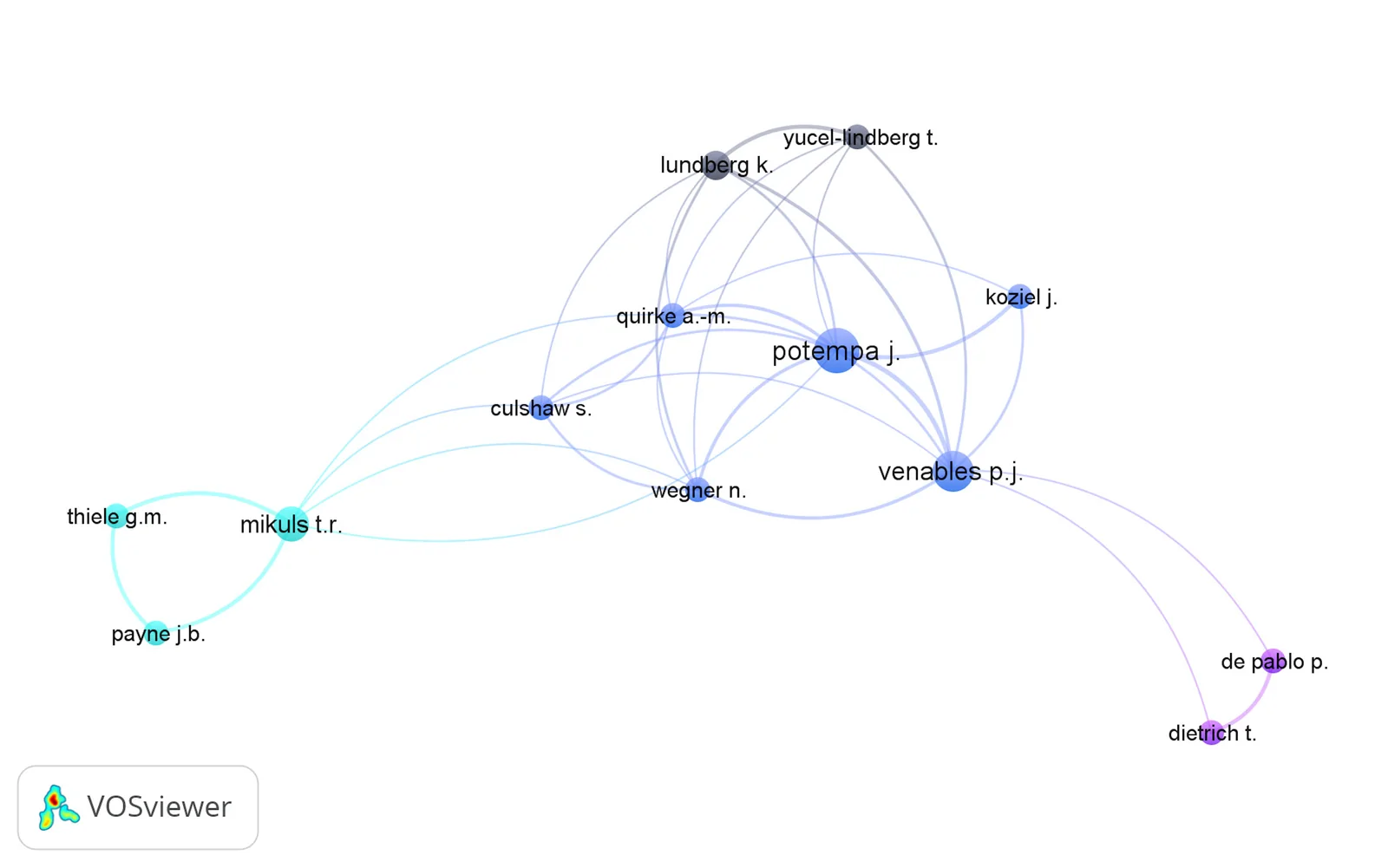
Fig. 3: Author density map and co-authorship network for the 100 most-cited articles. The figure illustrates the interconnections among different author groups. A minimum threshold of four publications per author was applied. Names in bold and represented by larger circles indicate the most prominent authors.

**Figure 4:**
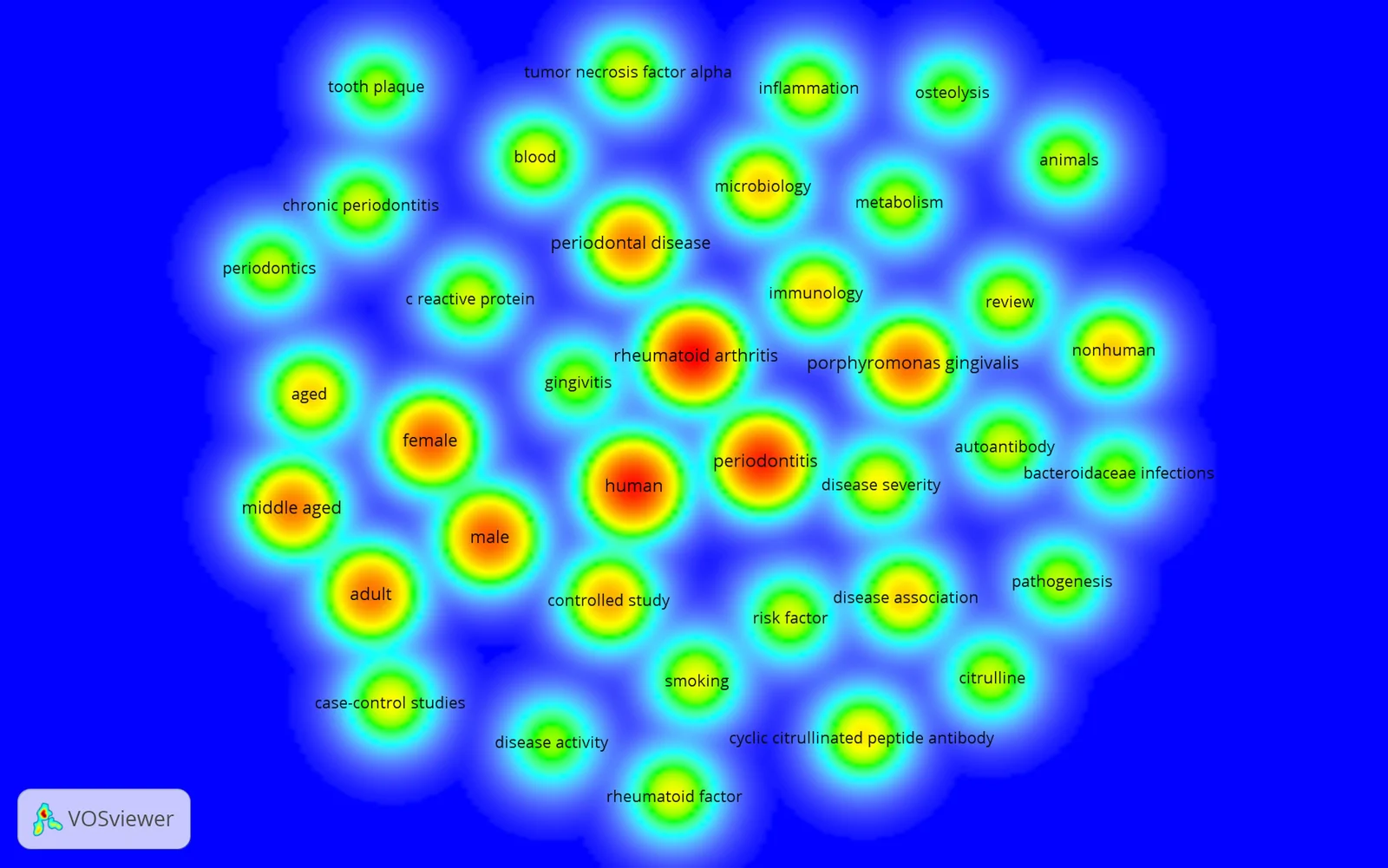
Fig. 4: Density map illustrating the distribution of keywords among the 100 most-cited articles. A minimum of five occurrences per term was set as the inclusion criterion. In the network analysis, keywords with higher frequency or stronger correlations are represented by larger nodes and warm colors (red, orange and yellow). Conversely, less frequent terms are displayed as smaller nodes and cooler colors (green and blue).

**Figure 5:**
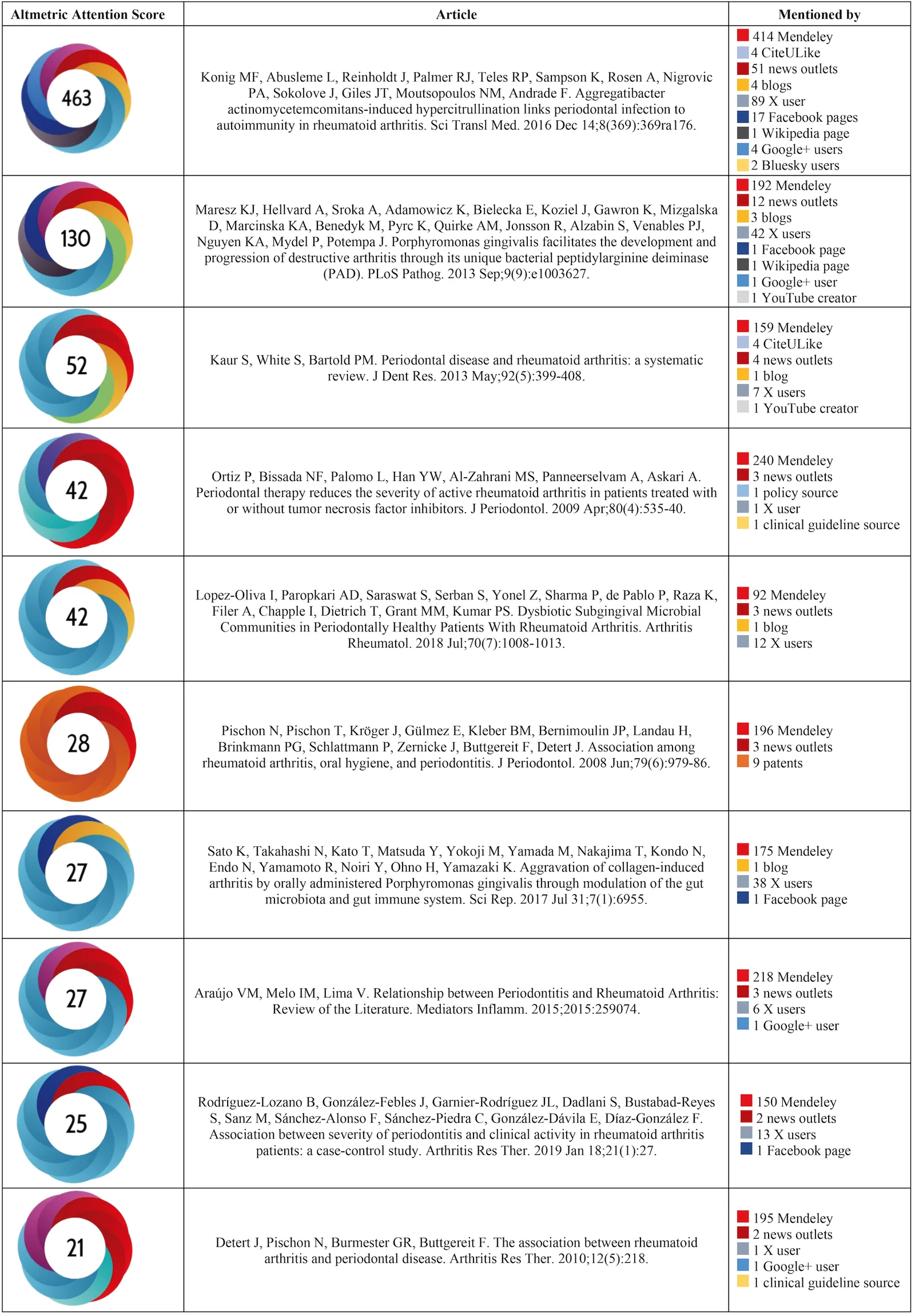
Fig. 5: The 10 articles with the highest altmetric score among the 100 most-cited articles on the relationship between periodontal disease and rheumatoid arthritis. X users= users of the social media platform formerly known as Twitter.

## Data Availability

None.
