# Supplementary material for: Mapping scientific knowledge on the association between periodontal disease and rheumatoid arthritis: A bibliometric and altmetric analysis of the 100 most-cited articles
Source: Med Oral Patol Oral Cir Bucal. 2026 Apr 9;31(5):e685–96. doi: 10.4317/medoral.28134 (PMC13636455; doi:10.4317/medoral.28134)
Supplement: Supplementary file 1 [file medoral-31-05-e685-s001.pdf]

Supllementary Table I. Bibliographic characteristics of the 100 selected articles on the relationship between Periodontal Disease and Rheumatoid Arthritis.

Ranking	Citation, year	Title of article	Journal	Language	Number of authors	Corresponding author's institution	Country of the corresponding author	Continent	Full reference
1	Wegner et al., 2010	Peptidylarginine deiminase from Porphyromonas gingivalis citrullinates human fibrinogen and α-enolase: Implications for autoimmunity in rheumatoid Arthritis	Arthritis and Rheumatism	English	10	Kennedy Institute of Rheumatology	United Kingdom	Europe	Wegner N, Wait R, Sroka A, Eick S, Nguyen KA, Lundberg K, Kinloch A, Culshaw S, Potempa J, Venables PJ. Peptidylarginine deiminase from Porphyromonas gingivalis citrullinates human fibrinogen and α-enolase: implications for autoimmunity in rheumatoid arthritis. Arthritis Rheum. 2010 Sep;62(9):2662-72.
2	Konig et al., 2016	Aggregatibacter actinomycetemcomitans-induced hypercitrullination links periodontal infection to autoimmunity in rheumatoid arthritis	Science Translational Medicine	English	12	Johns Hopkins University	United States	America	Konig MF, Abusleme L, Reinholdt J, Palmer RJ, Teles RP, Sampson K, Rosen A, Nigrovic PA, Sokolove J, Giles JT, Moutsopoulos NM, Andrade F. Aggregatibacter actinomycetemcomitans-induced hypercitrullination links periodontal infection to autoimmunity in rheumatoid arthritis. Sci Transl Med. 2016 Dec 14;8(369):369ra176.
3	Scher et al., 2012	Periodontal disease and the oral microbiota in new-onset	Arthritis and Rheumatism	English	14	New York University	United States	America	Scher JU, Ubeda C, Equinda M, Khanin R, Buischi Y, Viale A, Lipuma L, Attur M, Pillinger MH, Weissmann G, Littman DR, Pamer EG, Bretz WA, Abramson SB. Periodontal disease and the oral microbiota in new-onset

		rheumatoid arthritis				Hospital for Joint Diseases			rheumatoid arthritis. Arthritis Rheum. 2012 Oct;64(10):3083-94.
4	De Pablo et al., 2009	Periodontitis in systemic rheumatic diseases	Nature Reviews Rheumatology	English	4	University of Birmingham	United Kingdom	Europe	de Pablo P, Chapple IL, Buckley CD, Dietrich T. Periodontitis in systemic rheumatic diseases. Nat Rev Rheumatol. 2009 Apr;5(4):218-24.
5	De Pablo, Dietrich and McAlindon, 2008	Association of periodontal disease and tooth loss with rheumatoid arthritis in the US population	Journal of Rheumatology	English	3	Tufts-New England Medical Center	United States	America	de Pablo P, Dietrich T, McAlindon TE. Association of periodontal disease and tooth loss with rheumatoid arthritis in the US population. J Rheumatol. 2008 Jan;35(1):70-6.
6	Potempa, Mydel and Koziel, 2017	The case for periodontitis in the pathogenesis of rheumatoid arthritis	Nature Reviews Rheumatology	English	3	University of Louisville	United States	America	Potempa J, Mydel P, Koziel J. The case for periodontitis in the pathogenesis of rheumatoid arthritis. Nat Rev Rheumatol. 2017 Oct;13(10):606-620.
7	Mercado et al., 2001	Relationship between rheumatoid arthritis and periodontitis	Journal of Periodontology	English	4	University of Queensland	Australia	Oceania	Mercado FB, Marshall RI, Klestov AC, Bartold PM. Relationship between rheumatoid arthritis and periodontitis. J Periodontol. 2001 Jun;72(6):779-87.
8	Mikuls et al., 2014	Periodontitis and porphyromonas gingivalis in patients with	Arthritis and Rheumatology	English	22	University of Nebraska	United States	America	Mikuls TR, Payne JB, Yu F, Thiele GM, Reynolds RJ, Cannon GW, Markt J, McGowan D, Kerr GS, Redman RS, Reimold A, Griffiths G, Beatty M, Gonzalez SM, Bergman DA, Hamilton BC 3rd, Erickson AR, Sokolove J, Robinson WH, Walker C, Chandad F, O'Dell JR.

		rheumatoid arthritis							Periodontitis and Porphyromonas gingivalis in patients with rheumatoid arthritis. Arthritis Rheumatol. 2014 May;66(5):1090-100.
9	Rosenstein et al., 2004	Hypothesis: The humoral immune response to oral bacteria provides a stimulus for the development of rheumatoid arthritis	Inflammation	English	4	Center for Rheumatic and Autoimmune Diseases	United States	America	Rosenstein ED, Greenwald RA, Kushner LJ, Weissmann G. Hypothesis: the humoral immune response to oral bacteria provides a stimulus for the development of rheumatoid arthritis. Inflammation. 2004 Dec;28(6):311-8.
10	Pischon et al., 2008	Association among rheumatoid arthritis, oral hygiene, and periodontitis	Journal of Periodontology	English	12	Charité - Universitätsmedizin Berlin	Germany	Europe	Pischon N, Pischon T, Kröger J, Gülmez E, Kleber BM, Bernimoulin JP, Landau H, Brinkmann PG, Schlattmann P, Zernicke J, Buttgereit F, Detert J. Association among rheumatoid arthritis, oral hygiene, and periodontitis. J Periodontol. 2008 Jun;79(6):979-86.
11	Lundberg et al., 2010	Periodontitis in RA-the citrullinated enolase connection	Nature Reviews Rheumatology	English	4	Kennedy Institute of Rheumatology	United Kingdom	Europe	Lundberg K, Wegner N, Yucel-Lindberg T, Venables PJ. Periodontitis in RA-the citrullinated enolase connection. Nat Rev Rheumatol. 2010 Dec;6(12):727-30.
12	Ortiz et al., 2009	Periodontal therapy reduces the seveity of active reeumatoid arthritis in patients treated with or without	Journal of Periodontology	English	7	Case Western Reserve University	United States	America	Ortiz P, Bissada NF, Palomo L, Han YW, Al-Zahrani MS, Panneerselvam A, Askari A. Periodontal therapy reduces the severity of active rheumatoid arthritis in patients treated with or without tumor necrosis factor inhibitors. J Periodontol. 2009 Apr;80(4):535-40.

		tumor necrosis factor inhibitors							
13	Mercado et al., 2000	Is there a relationship between rheumatoid arthritis and periodontal disease?	Journal of Clinical Periodontology	English	4	University of Queensland	Australia	Oceania	Mercado F, Marshall RI, Klestov AC, Bartold PM. Is there a relationship between rheumatoid arthritis and periodontal disease? J Clin Periodontol. 2000 Apr;27(4):267-72.
14	Bartold, Marshall and Haynes, 2005	Periodontitis and rheumatoid arthritis: A review	Journal of Periodontology	English	3	University of Adelaide	Australia	Oceania	Bartold PM, Marshall RI, Haynes DR. Periodontitis and rheumatoid arthritis: a review. J Periodontol. 2005 Nov;76(11 Suppl):2066-74.
15	Mikuls et al., 2009	Antibody responses to Porphyromonas gingivalis (P. gingivalis) in subjects with rheumatoid arthritis and periodontitis	International Immunopharmacology	English	9	University of Nebraska	United States	America	Mikuls TR, Payne JB, Reinhardt RA, Thiele GM, Maziarz E, Cannella AC, Holers VM, Kuhn KA, O'Dell JR. Antibody responses to Porphyromonas gingivalis (P. gingivalis) in subjects with rheumatoid arthritis and periodontitis. Int Immunopharmacol. 2009 Jan;9(1):38-42.
16	Dissick et al., 2010	Association of periodontitis with rheumatoid arthritis: A pilot study	Journal of Periodontology	English	10	Washington Veterans Affairs Medical Center	United States	America	Dissick A, Redman RS, Jones M, Rangan BV, Reimold A, Griffiths GR, Mikuls TR, Amdur RL, Richards JS, Kerr GS. Association of periodontitis with rheumatoid arthritis: a pilot study. J Periodontol. 2010 Feb;81(2):223-30.

17	Maresz et al., 2013	Porphyromonas gingivalis Facilitates the Development and Progression of Destructive Arthritis through Its Unique Bacterial Peptidylarginine Deiminase (PAD)	Plos Pathogens	English	18	University of Bergen	Norway	Europe	Maresz KJ, Hellvard A, Sroka A, Adamowicz K, Bielecka E, Koziel J, Gawron K, Mizgalska D, Marcinska KA, Benedyk M, Pyrc K, Quirke AM, Jonsson R, Alzabin S, Venables PJ, Nguyen KA, Mydel P, Potempa J. Porphyromonas gingivalis facilitates the development and progression of destructive arthritis through its unique bacterial peptidylarginine deiminase (PAD). PLoS Pathog. 2013 Sep;9(9):e1003627.
18	Kaur, White and Bartold, 2013	Periodontal disease and rheumatoid arthritis: A systematic review	Journal of Dental Research	English	3	University of Adelaide	Australia	Oceania	Kaur S, White S, Bartold PM. Periodontal disease and rheumatoid arthritis: a systematic review. J Dent Res. 2013 May;92(5):399-408.
19	Mikuls et al., 2012	Porphyromonas gingivalis and disease-related autoantibodies in individuals at increased risk of rheumatoid arthritis	Arthritis and Rheumatism	English	15	University of Nebraska	United States	America	Mikuls TR, Thiele GM, Deane KD, Payne JB, O'Dell JR, Yu F, Sayles H, Weisman MH, Gregersen PK, Buckner JH, Keating RM, Derber LA, Robinson WH, Holers VM, Norris JM. Porphyromonas gingivalis and disease-related autoantibodies in individuals at increased risk of rheumatoid arthritis. Arthritis Rheum. 2012 Nov;64(11):3522-30.
20	Hitchon et al., 2010	Antibodies to Porphyromonas gingivalis are associated with anticitrullinated protein antibodies	Journal of Rheumatolo gy	English	13	University of Manitoba	Canada	America	Hitchon CA, Chandad F, Ferucci ED, Willemze A, Ioan-Facsinay A, van der Woude D, Markland J, Robinson D, Elias B, Newkirk M, Toes RM, Huizinga TW, El-Gabalawy HS. Antibodies to porphyromonas gingivalis are associated with anticitrullinated protein antibodies in

		in patients with rheumatoid arthritis and their relatives							patients with rheumatoid arthritis and their relatives. J Rheumatol. 2010 Jun;37(6):1105-12.
21	Martinez - Martinez et al., 2009	Detection of periodontal bacterial DNA in serum and synovial fluid in refractory rheumatoid arthritis patients	Journal of Clinical Periodontology	English	6	San Luis Potosi University	Mexico	America	Martinez-Martinez RE, Abud-Mendoza C, Patiño-Marin N, Rizo-Rodríguez JC, Little JW, Loyola-Rodríguez JP. Detection of periodontal bacterial DNA in serum and synovial fluid in refractory rheumatoid arthritis patients. J Clin Periodontol. 2009 Dec;36(12):1004-10.
22	Detert et al., 2010	The association between rheumatoid arthritis and periodontal disease	Arthritis Research and Therapy	English	4	Charité - Universitätsmedizin Berlin	Germany	Europe	Detert J, Pischon N, Burmester GR, Buttgereit F. The association between rheumatoid arthritis and periodontal disease. Arthritis Res Ther. 2010;12(5):218.
23	Al-Katma et al., 2007	Control of periodontal infection reduces the severity of active rheumatoid arthritis	Journal of Clinical Rheumatology	English	5	University Hospitals of Cleveland	United States	America	Al-Katma MK, Bissada NF, Bordeaux JM, Sue J, Askari AD. Control of periodontal infection reduces the severity of active rheumatoid arthritis. J Clin Rheumatol. 2007 Jun;13(3):134-7.
24	De Smit et al., 2012	Periodontitis in established rheumatoid arthritis patients: a cross-sectional	Arthritis Research and Therapy	English	6	University of Groningen	The Netherlands	Europe	de Smit M, Westra J, Vissink A, Doornbos-van der Meer B, Brouwer E, van Winkelhoff AJ. Periodontitis in established rheumatoid arthritis patients: a cross-

		clinical, microbiological and serological study							sectional clinical, microbiological and serological study. Arthritis Res Ther. 2012 Oct 17;14(5):R222.
25	Koziel, Mydel and Potempa, 2014	The link between periodontal disease and rheumatoid arthritis: An updated review	Current Rheumatology Reports	English	3	Jagiellonian University	Poland	Europe	Koziel J, Mydel P, Potempa J. The link between periodontal disease and rheumatoid arthritis: an updated review. Curr Rheumatol Rep. 2014 Mar;16(3):408.
26	Fuggle et al., 2016	Hand to mouth: A systematic review and meta-analysis of the association between rheumatoid arthritis and periodontitis	Frontiers in Immunology	English	4	St George's University of London	United Kingdom	Europe	Fuggle NR, Smith TO, Kaul A, Sofat N. Hand to Mouth: A Systematic Review and Meta-Analysis of the Association between Rheumatoid Arthritis and Periodontitis. Front Immunol. 2016 Mar 2;7:80.
27	Quirke et al., 2014	Heightened immune response to autocitrullinated Porphyromonas gingivalis peptidylarginine deiminase: A potential mechanism for breaching immunologic	Annals of the Rheumatic Diseases	English	15	Kennedy Institute of Rheumatology	United Kingdom	Europe	Quirke AM, Lugli EB, Wegner N, Hamilton BC, Charles P, Chowdhury M, Ytterberg AJ, Zubarev RA, Potempa J, Culshaw S, Guo Y, Fisher BA, Thiele G, Mikuls TR, Venables PJ. Heightened immune response to autocitrullinated Porphyromonas gingivalis peptidylarginine deiminase: a potential mechanism for breaching immunologic tolerance in rheumatoid arthritis. Ann Rheum Dis. 2014 Jan;73(1):263-9.

		tolerance in rheumatoid arthritis							
28	Mercado, Marshall and Bartold, 2003	Inter-relationships between rheumatoid arthritis and periodontal disease: A review	Journal of Clinical Periodontology	English	3	University of Adelaide	Australia	Oceania	Mercado FB, Marshall RI, Bartold PM. Inter-relationships between rheumatoid arthritis and periodontal disease. A review. J Clin Periodontol. 2003 Sep;30(9):761-72.
29	Sato et al., 2017	Aggravation of collagen-induced arthritis by orally administered Porphyromonas gingivalis through modulation of the gut microbiota and gut immune system	Scientific Reports	English	13	Niigata University	Japan	Asia	Sato K, Takahashi N, Kato T, Matsuda Y, Yokoji M, Yamada M, Nakajima T, Kondo N, Endo N, Yamamoto R, Noiri Y, Ohno H, Yamazaki K. Aggravation of collagen-induced arthritis by orally administered Porphyromonas gingivalis through modulation of the gut microbiota and gut immune system. Sci Rep. 2017 Jul 31;7(1):6955.
30	Aquino et al., 2014	Periodontal pathogens directly promote autoimmune experimental arthritis by inducing a TLR2- and IL-1-Driven Th17 Response	Journal of Immunology	English	14	Radboud University Nijmegen Medical Centre	Netherlands	Europe	de Aquino SG, Abdollahi-Roodsaz S, Koenders MI, van de Loo FA, Pruijn GJ, Marijnissen RJ, Walgreen B, Helsen MM, van den Bersselaar LA, de Molon RS, Avila Campos MJ, Cunha FQ, Cirelli JA, van den Berg WB. Periodontal pathogens directly promote autoimmune experimental arthritis by inducing a TLR2- and IL-1-driven Th17 response. J Immunol. 2014 May 1;192(9):4103-11.

31	Kässer et al., 1997	Risk for periodontal disease in patients with longstanding rheumatoid arthritis	Arthritis and Rheumatism	English	6	Clinic for Rheumatic Diseases II	Germany	Europe	Kässer UR, Gleissner C, Dehne F, Michel A, Willershausen-Zönnchen B, Bolten WW. Risk for periodontal disease in patients with longstanding rheumatoid arthritis. Arthritis Rheum. 1997 Dec;40(12):2248-51
32	De Molon et al., 2019	Linkage of periodontitis and rheumatoid arthritis: Current evidence and potential biological interactions	International Journal of Molecular Sciences	English	5	Sao Paulo State University	Brazil	America	de Molon RS, Rossa C Jr, Thurlings RM, Cirelli JA, Koenders MI. Linkage of Periodontitis and Rheumatoid Arthritis: Current Evidence and Potential Biological Interactions. Int J Mol Sci. 2019 Sep 13;20(18):4541.
33	Chen et al., 2013	Association between a history of periodontitis and the risk of rheumatoid arthritis: A nationwide, population-based, case-control study	Annals of the Rheumatic Diseases	English	11	Taichung Veterans General Hospital	Taiwan	Asia	Chen HH, Huang N, Chen YM, Chen TJ, Chou P, Lee YL, Chou YJ, Lan JL, Lai KL, Lin CH, Chen DY. Association between a history of periodontitis and the risk of rheumatoid arthritis: a nationwide, population-based, case-control study. Ann Rheum Dis. 2013 Jul;72(7):1206-11.
34	Mayer, Balbir-Gurman and Machtei, 2009	Anti-tumor necrosis factor-alpha therapy and periodontal parameters in patients with	Journal of Periodontology	English	3	Rambam Health Care Campus	Israel	Asia	Mayer Y, Balbir-Gurman A, Machtei EE. Anti-tumor necrosis factor-alpha therapy and periodontal parameters in patients with rheumatoid arthritis. J Periodontol. 2009 Sep;80(9):1414-20.

		rheumatoid arthritis							
35	Marotte et al., 2006	The association between periodontal disease and joint destruction in rheumatoid arthritis extends the link between the HLA-DR shared epitope and severity of bone destruction	Annals of the Rheumatic Diseases	English	6	Hôpital Edouard Hérriot	France	Europe	Marotte H, Farge P, Gaudin P, Alexandre C, Mougin B, Miossec P. The association between periodontal disease and joint destruction in rheumatoid arthritis extends the link between the HLA-DR shared epitope and severity of bone destruction. Ann Rheum Dis. 2006 Jul;65(7):905-9.
36	Bingham and Moni, 2013	Periodontal disease and rheumatoid arthritis: The evidence accumulates for complex pathobiologic interactions	Current Opinion in Rheumatolo gy	English	2	Johns Hopkins University	United States	America	Bingham CO 3rd, Moni M. Periodontal disease and rheumatoid arthritis: the evidence accumulates for complex pathobiologic interactions. Curr Opin Rheumatol. 2013 May;25(3):345-53.
37	Ribeiro, Leão and Novaes, 2005	Periodontal infection as a possible severity factor for rheumatoid arthritis	Journal of Clinical Periodontolo gy	English	3	University of Sao Paulo	Brazil	America	Ribeiro J, Leão A, Novaes AB. Periodontal infection as a possible severity factor for rheumatoid arthritis. J Clin Periodontol. 2005 Apr;32(4):412-6.

38	Kharlamova et al., 2016	Antibodies to Porphyromonas gingivalis Indicate Interaction between Oral Infection, Smoking, and Risk Genes in Rheumatoid Arthritis Etiology	Arthritis and Rheumatology	English	12	Karolinska Institutet	Sweden	Europe	Kharlamova N, Jiang X, Sherina N, Potempa B, Israelsson L, Quirke AM, Eriksson K, Yucel-Lindberg T, Venables PJ, Potempa J, Alfredsson L, Lundberg K. Antibodies to Porphyromonas gingivalis Indicate Interaction Between Oral Infection, Smoking, and Risk Genes in Rheumatoid Arthritis Etiology. Arthritis Rheumatol. 2016 Mar;68(3):604-13.
39	Harvey et al., 2013	Expression of peptidylarginine deiminase-2 and -4, citrullinated proteins and anti-citrullinated protein antibodies in human gingiva	Journal of Periodontal Research	English	6	University of Adelaide	Australia	Oceania	Harvey GP, Fitzsimmons TR, Dhamarpatni AA, Marchant C, Haynes DR, Bartold PM. Expression of peptidylarginine deiminase-2 and -4, citrullinated proteins and anti-citrullinated protein antibodies in human gingiva. J Periodontal Res. 2013 Apr;48(2):252-61.
40	Nesse et al., 2012	The periodontium of periodontitis patients contains citrullinated proteins which may play a role in ACPA (anti-citrullinated protein antibody) formation	Journal of Clinical Periodontology	English	7	University of Groningen	Netherlands	Europe	Nesse W, Westra J, van der Wal JE, Abbas F, Nicholas AP, Vissink A, Brouwer E. The periodontium of periodontitis patients contains citrullinated proteins which may play a role in ACPA (anti-citrullinated protein antibody) formation. J Clin Periodontol. 2012 Jul;39(7):599-607.

41	Persson, 2012	Rheumatoid arthritis and periodontitis - inflammatory and infectious connections. Review of the literature	Journal of Oral Microbiology	English	1	University of Kristianstad	Sweden	Europe	Rutger Persson G. Rheumatoid arthritis and periodontitis - inflammatory and infectious connections. Review of the literature. J Oral Microbiol. 2012;4.
42	Pers et al., 2008	Anti-TNF-α immunotherapy is associated with increased gingival inflammation without clinical attachment loss in subjects with rheumatoid arthritis	Journal of Periodontology	English	4	University of Brest	France	Europe	Pers JO, Saraux A, Pierre R, Youinou P. Anti-TNF-alpha immunotherapy is associated with increased gingival inflammation without clinical attachment loss in subjects with rheumatoid arthritis. J Periodontol. 2008 Sep;79(9):1645-51.
43	Araújo, Melo and Lima, 2015	Relationship between periodontitis and rheumatoid arthritis: Review of the literature	Mediators of Inflammation	English	3	Federal University of Ceara	Brazil	America	Araújo VM, Melo IM, Lima V. Relationship between Periodontitis and Rheumatoid Arthritis: Review of the Literature. Mediators Inflamm. 2015;2015:259074.
44	Demmer et al., 2011	Periodontal disease, tooth loss and incident rheumatoid arthritis: Results from the First	Journal of Clinical Periodontology	English	4	Columbia University	United States	America	Demmer RT, Molitor JA, Jacobs DR Jr, Michalowicz BS. Periodontal disease, tooth loss and incident rheumatoid arthritis: results from the First National Health and Nutrition Examination Survey and its epidemiological

		National Health and Nutrition Examination Survey and its epidemiological follow-up study							follow-up study. J Clin Periodontol. 2011 Nov;38(11):998-1006.
45	Rodríguez-Lozano et al., 2019	Association between severity of periodontitis and clinical activity in rheumatoid arthritis patients: A case-control study	Arthritis Research and Therapy	English	10	Hospital Universitario de Canarias	Spain	Europe	Rodríguez-Lozano B, González-Febles J, Garnier-Rodríguez JL, Dadlani S, Bustabad-Reyes S, Sanz M, Sánchez-Alonso F, Sánchez-Piedra C, González-Dávila E, Díaz-González F. Association between severity of periodontitis and clinical activity in rheumatoid arthritis patients: a case-control study. Arthritis Res Ther. 2019 Jan 18;21(1):27.
46	Kaur et al., 2014	Does periodontal treatment influence clinical and biochemical measures for rheumatoid arthritis? A systematic review and meta-analysis	Seminars in Arthritis and Rheumatism	English	4	University of Adelaide	Australia	Oceania	Kaur S, Bright R, Proudman SM, Bartold PM. Does periodontal treatment influence clinical and biochemical measures for rheumatoid arthritis? A systematic review and meta-analysis. Semin Arthritis Rheum. 2014 Oct;44(2):113-22.
47	Ogrendik et al., 2005	Serum antibodies to oral anaerobic bacteria in patients with	Medgenmed Medscape General Medicine	English	5	Nazilli State Hospital	Turkey	Asia	Ogrendik M, Kokino S, Ozdemir F, Bird PS, Hamlet S. Serum antibodies to oral anaerobic bacteria in patients with rheumatoid arthritis. MedGenMed. 2005 Jun 16;7(2):2.

		rheumatoid arthritis							
48	Ishida et al., 2012	Interleukin-6 gene promoter methylation in rheumatoid arthritis and chronic periodontitis	Journal of Periodontology	English	9	Niigata University	Japan	Asia	Ishida K, Kobayashi T, Ito S, Komatsu Y, Yokoyama T, Okada M, Abe A, Murasawa A, Yoshie H. Interleukin-6 gene promoter methylation in rheumatoid arthritis and chronic periodontitis. J Periodontol. 2012 Jul;83(7):917-25.
49	Lappin et al., 2013	Influence of periodontal disease, Porphyromonas gingivalis and cigarette smoking on systemic anti-citrullinated peptide antibody titres	Journal of Clinical Periodontology	English	10	University of Glasgow	United Kingdom	Europe	Lappin DF, Apatzidou D, Quirke AM, Oliver-Bell J, Butcher JP, Kinane DF, Riggio MP, Venables P, McInnes IB, Culshaw S. Influence of periodontal disease, Porphyromonas gingivalis and cigarette smoking on systemic anti-citrullinated peptide antibody titres. J Clin Periodontol. 2013 Oct;40(10):907-15.
50	Berthelot and Le Goff, 2010	Rheumatoid arthritis and periodontal disease	Joint Bone Spine	English	2	CHU de Nantes	France	Europe	Berthelot JM, Le Goff B. Rheumatoid arthritis and periodontal disease. Joint Bone Spine. 2010 Dec;77(6):537-41.
51	Kassem et al., 2015	Porphyromonas gingivalis stimulates bone resorption by enhancing RANKL (receptor	Journal of Biological Chemistry	English	6	Umeå University	Sweden	Europe	Kassem A, Henning P, Lundberg P, Souza PP, Lindholm C, Lerner UH. Porphyromonas gingivalis Stimulates Bone Resorption by Enhancing RANKL (Receptor Activator of NF-κB Ligand) through Activation of Toll-

		activator of NF-κB ligand) through activation of toll-like receptor 2 in osteoblasts							like Receptor 2 in Osteoblasts. J Biol Chem. 2015 Aug 14;290(33):20147-58.
52	Joseph et al., 2013	Association between chronic periodontitis and rheumatoid arthritis: A hospital-based case-control study	Rheumatology International	English	4	Government Dental College	India	Asia	Joseph R, Rajappan S, Nath SG, Paul BJ. Association between chronic periodontitis and rheumatoid arthritis: a hospital-based case-control study. Rheumatol Int. 2013 Jan;33(1):103-9.
53	Cheng et al., 2017	Periodontal disease and periodontal bacteria as triggers for rheumatoid arthritis	Best Practice and Research Clinical Rheumatology	English	5	University of Leeds	United Kingdom	Europe	Cheng Z, Meade J, Mankia K, Emery P, Devine DA. Periodontal disease and periodontal bacteria as triggers for rheumatoid arthritis. Best Pract Res Clin Rheumatol. 2017 Feb;31(1):19-30.
54	Cantley et al., 2011	Pre-existing periodontitis exacerbates experimental arthritis in a mouse model	Journal of Clinical Periodontology	English	4	University of Adelaide	Australia	Oceania	Cantley MD, Haynes DR, Marino V, Bartold PM. Pre-existing periodontitis exacerbates experimental arthritis in a mouse model. J Clin Periodontol. 2011 Jun;38(6):532-41.

55	Okada et al., 2013	Periodontal treatment decreases levels of antibodies to porphyromonas gingivalis and citrulline in patients with rheumatoid arthritis and periodontitis	Journal of Periodontology	English	7	Niigata University	Japan	Asia	Okada M, Kobayashi T, Ito S, Yokoyama T, Abe A, Murasawa A, Yoshie H. Periodontal treatment decreases levels of antibodies to Porphyromonas gingivalis and citrulline in patients with rheumatoid arthritis and periodontitis. J Periodontol. 2013 Dec;84(12):e74-84.
56	Mirrieless et al., 2010	Rheumatoid arthritis and salivary Biomarkers of periodontal disease	Journal of Clinical Periodontology	English	7	University of Kentucky	United States	America	Mirrielees J, Crofford LJ, Lin Y, Kryscio RJ, Dawson DR 3rd, Ebersole JL, Miller CS. Rheumatoid arthritis and salivary Biomarkers of periodontal disease. J Clin Periodontol. 2010 Dec;37(12):1068-74.
57	Marchesan et al., 2013	Porphyromonas gingivalis oral infection exacerbates the development and severity of collagen-induced arthritis	Arthritis Research and Therapy	English	13	University of Michigan	United States	America	Marchesan JT, Gerow EA, Schaff R, Taut AD, Shin SY, Sugai J, Brand D, Burberry A, Jorns J, Lundy SK, Nuñez G, Fox DA, Giannobile WV. Porphyromonas gingivalis oral infection exacerbates the development and severity of collagen-induced arthritis. Arthritis Res Ther. 2013 Nov 12;15(6):R186.
58	Reichert et al., 2013	Detection of oral bacterial DNA in synovial fluid	Journal of Clinical Periodontology	English	10	Martin-Luther University Halle-Wittenberg	Germany	Europe	Reichert S, Haffner M, Keyßer G, Schäfer C, Stein JM, Schaller HG, Wienke A, Strauss H, Heide S, Schulz S. Detection of oral bacterial DNA in synovial fluid. J Clin Periodontol. 2013 Jun;40(6):591-8.

59	Corrêa et al., 2019	Oral microbial dysbiosis linked to worsened periodontal condition in rheumatoid arthritis patients	Scientific Reports	English	14	University of Pennsylvania	United States	America	Corrêa JD, Fernandes GR, Calderaro DC, Mendonça SMS, Silva JM, Albiero ML, Cunha FQ, Xiao E, Ferreira GA, Teixeira AL, Mukherjee C, Leys EJ, Silva TA, Graves DT. Oral microbial dysbiosis linked to worsened periodontal condition in rheumatoid arthritis patients. Sci Rep. 2019 Jun 10;9(1):8379.
60	Gómez-Bañuelos et al., 2019	Rheumatoid arthritis-associated mechanisms of porphyromonas gingivalis and aggregatibacter actinomycetemcomitans	Journal of Clinical Medicine	English	4	Johns Hopkins University	United States	America	Gómez-Bañuelos E, Mukherjee A, Darrah E, Andrade F. Rheumatoid Arthritis-Associated Mechanisms of Porphyromonas gingivalis and Aggregatibacter actinomycetemcomitans. J Clin Med. 2019 Aug 26;8(9):1309.
61	Laugisch et al., 2016	Citrullination in the periodontium—a possible link between periodontitis and rheumatoid arthritis	Clinical Oral Investigations	English	11	University of Bern	Switzerland	Europe	Laugisch O, Wong A, Sroka A, Kantyka T, Koziel J, Neuhaus K, Sculean A, Venables PJ, Potempa J, Möller B, Eick S. Citrullination in the periodontium--a possible link between periodontitis and rheumatoid arthritis. Clin Oral Investig. 2016 May;20(4):675-83.
62	Mankia et al., 2019	Prevalence of Periodontal Disease and Periodontopathic Bacteria in Anti-Cyclic	JAMA Network Open	English	13	University of Leeds	United Kingdom	Europe	Mankia K, Cheng Z, Do T, Hunt L, Meade J, Kang J, Clerehugh V, Speirs A, Tugnait A, Hensor EMA, Nam JL, Devine DA, Emery P. Prevalence of Periodontal Disease and Periodontopathic Bacteria in Anti-Cyclic Citrullinated Protein Antibody-Positive At-Risk Adults

		Citrullinated Protein Antibody-Positive At-Risk Adults Without Arthritis							Without Arthritis. JAMA Netw Open. 2019 Jun 5;2(6):e195394.
63	González-Febles and Sanz, 2021	Periodontitis and rheumatoid arthritis: What have we learned about their connection and their treatment?	Periodontology 2000	English	2	University Complutense of Madrid	Spain	Europe	González-Febles J, Sanz M. Periodontitis and rheumatoid arthritis: What have we learned about their connection and their treatment? Periodontol 2000. 2021 Oct;87(1):181-203.
64	Mangat et al., 2010	Bacterial and human peptidylarginine deiminases: Targets for inhibiting the autoimmune response in rheumatoid arthritis?	Arthritis Research and Therapy	English	4	Kennedy Institute of Rheumatology	United Kingdom	Europe	Mangat P, Wegner N, Venables PJ, Potempa J. Bacterial and human peptidylarginine deiminases: targets for inhibiting the autoimmune response in rheumatoid arthritis? Arthritis Res Ther. 2010;12(3):209.
65	Liao et al., 2009	Porphyromonas gingivalis may play an important role in the pathogenesis of periodontitis-associated	Medical Hypotheses	English	6	Wuhan University	China	Asia	Liao F, Li Z, Wang Y, Shi B, Gong Z, Cheng X. Porphyromonas gingivalis may play an important role in the pathogenesis of periodontitis-associated rheumatoid arthritis. Med Hypotheses. 2009 Jun;72(6):732-5.

		rheumatoid arthritis							
66	Arkema, Karlson and Costenbader, 2010	A prospective study of periodontal disease and risk of rheumatoid arthritis	Journal of Rheumatology	English	3	Harvard University	United States	America	Arkema EV, Karlson EW, Costenbader KH. A prospective study of periodontal disease and risk of rheumatoid arthritis. J Rheumatol. 2010 Sep;37(9):1800-4.
67	Konig et al., 2015	Defining the role of Porphyromonas gingivalis peptidylarginine deiminase (PPAD) in rheumatoid arthritis through the study of PPAD biology	Annals of the Rheumatic Diseases	English	5	Johns Hopkins University	United States	America	Konig MF, Paracha AS, Moni M, Bingham CO 3rd, Andrade F. Defining the role of Porphyromonas gingivalis peptidylarginine deiminase (PPAD) in rheumatoid arthritis through the study of PPAD biology. Ann Rheum Dis. 2015 Nov;74(11):2054-61
68	Kriebel et al., 2018	Oral biofilms from symbiotic to pathogenic interactions and associated disease - Connection of periodontitis and rheumatic arthritis by peptidylarginine deiminase	Frontiers in Microbiology	English	5	University of Rostock	Germany	Europe	Kriebel K, Hieke C, Müller-Hilke B, Nakata M, Kreikemeyer B. Oral Biofilms from Symbiotic to Pathogenic Interactions and Associated Disease - Connection of Periodontitis and Rheumatic Arthritis by Peptidylarginine Deiminase. Front Microbiol. 2018 Jan 30;9:53.

69	Queiroz-Junior et al., 2011	Experimental Arthritis Triggers Periodontal Disease in Mice: Involvement of TNF-α and the Oral Microbiota	Journal of Immunology	English	10	Federal University of Minas Gerais	Brazil	America	Queiroz-Junior CM, Madeira MF, Coelho FM, Costa VV, Bessoni RL, Sousa LF, Garlet GP, Souza Dda G, Teixeira MM, Silva TA. Experimental arthritis triggers periodontal disease in mice: involvement of TNF-α and the oral Microbiota. J Immunol. 2011 Oct 1;187(7):3821-30.
70	De Pablo et al., 2014	The autoantibody repertoire in periodontitis: A role in the induction of autoimmunity to citrullinated proteins in rheumatoid arthritis?	Annals of the Rheumatic Diseases	English	8	University of Birmingham	United Kingdom	Europe	de Pablo P, Dietrich T, Chapple IL, Milward M, Chowdhury M, Charles PJ, Buckley CD, Venables PJ. The autoantibody repertoire in periodontitis: a role in the induction of autoimmunity to citrullinated proteins in rheumatoid arthritis? Ann Rheum Dis. 2014 Mar;73(3):580-6.
71	Leech and Bartold, 2015	The association between rheumatoid arthritis and periodontitis	Best Practice and Research Clinical Rheumatology	English	2	Monash Medical Centre	Australia	Oceania	Leech MT, Bartold PM. The association between rheumatoid arthritis and periodontitis. Best Pract Res Clin Rheumatol. 2015 Apr;29(2):189-201.
72	Erciyas et al., 2013	Effects of periodontal therapy on disease activity and systemic inflammation in	Oral Diseases	English	8	Gaziantep University	Turkey	Asia	Erciyas K, Sezer U, Ustün K, Pehlivan Y, Kisacik B, Senyurt SZ, Tarakçioğlu M, Onat AM. Effects of periodontal therapy on disease activity and systemic inflammation in rheumatoid arthritis patients. Oral Dis. 2013 May;19(4):394-400.

		rheumatoid arthritis patients							
73	Lopez-Oliva et al., 2018	Dysbiotic Subgingival Microbial Communities in Periodontally Healthy Patients with Rheumatoid Arthritis	Arthritis and Rheumatology	English	13	The Ohio State University	United States	America	Lopez-Oliva I, Paropkari AD, Saraswat S, Serban S, Yonel Z, Sharma P, de Pablo P, Raza K, Filer A, Chapple I, Dietrich T, Grant MM, Kumar PS. Dysbiotic Subgingival Microbial Communities in Periodontally Healthy Patients with Rheumatoid Arthritis. Arthritis Rheumatol. 2018 Jul;70(7):1008-1013.
74	Bartold and Lopez-Oliva, 2020	Periodontitis and rheumatoid arthritis: An update 2012-2017	Oral Diseases	English	2	University of Adelaide	Australia	Oceania	Bartold PM, Lopez-Oliva I. Periodontitis and rheumatoid arthritis: An update 2012-2017. Periodontol 2000. 2020 Jun;83(1):189-212.
75	Farquharson, Butcher and Culshaw, 2012	Periodontitis, Porphyromonas, and the pathogenesis of rheumatoid arthritis	Mucosal Immunology	English	3	University of Glasgow	United Kingdom	Europe	Farquharson D, Butcher JP, Culshaw S. Periodontitis, Porphyromonas, and the pathogenesis of rheumatoid arthritis. Mucosal Immunol. 2012 Mar;5(2):112-20.
76	Tolo and Jorkjend, 1990	Serum antibodies and loss of periodontal bone in patients with rheumatoid arthritis	Journal of Clinical Periodontology	English	2	University of Oslo	Norway	Europe	Tolo K, Jorkjend L. Serum antibodies and loss of periodontal bone in patients with rheumatoid arthritis. J Clin Periodontol. 1990 May;17(5):288-91.

77	Bartold et al., 2010	Effect of Porphyromonas gingivalis-induced inflammation on the development of rheumatoid arthritis	Journal of Clinical Periodontology	English	4	University of Adelaide	Australia	Oceania	Bartold PM, Marino V, Cantley M, Haynes DR. Effect of Porphyromonas gingivalis-induced inflammation on the development of rheumatoid arthritis. J Clin Periodontol. 2010 May;37(5):405-11.
78	Eriksson et al., 2019	Periodontal health and oral microbiota in patients with rheumatoid arthritis	Journal of Clinical Medicine	English	15	Karolinska Institutet	Sweden	Europe	Eriksson K, Fei G, Lundmark A, Benchimol D, Lee L, Hu YOO, Kats A, Saevarsdottir S, Catrina AI, Klinge B, Andersson AF, Klareskog L, Lundberg K, Jansson L, Yucel-Lindberg T. Periodontal Health and Oral Microbiota in Patients with Rheumatoid Arthritis. J Clin Med. 2019 May 8;8(5):630.
79	Pinho et al., 2009	Relationship between periodontitis and rheumatoid arthritis and the effect of non-surgical periodontal treatment	Brazilian Dental Journal	English	4	Pontifical Catholic University	Brazil	America	Pinho Mde N, Oliveira RD, Novaes AB Jr, Voltarelli JC. Relationship between periodontitis and rheumatoid arthritis and the effect of non-surgical periodontal treatment. Braz Dent J. 2009;20(5):355-64.
80	Payne et al., 2015	The Link Between Periodontitis and Rheumatoid Arthritis: A	Current Oral Health Reports	English	4	University of Nebraska	United States	America	Payne JB, Golub LM, Thiele GM, Mikuls TR. The Link Between Periodontitis and Rheumatoid Arthritis: A Periodontist's Perspective. Curr Oral Health Rep. 2015;2(1):20-29.

		Periodontist's Perspective							
81	Perricone et al., 2019	Porphyromonas gingivalis and rheumatoid arthritis	Current Opinion in Rheumatology	English	10	Sapienza University of Rome	Italy	Europe	Perricone C, Ceccarelli F, Saccucci M, Di Carlo G, Bogdanos DP, Lucchetti R, Pilloni A, Valesini G, Polimeni A, Conti F. Porphyromonas gingivalis and rheumatoid arthritis. Curr Opin Rheumatol. 2019 Sep;31(5):517-524.
82	Eriksson et al., 2016	Prevalence of periodontitis in patients with established rheumatoid arthritis: A swedish population based case-control study	Plos One	English	11	Karolinska Institutet	Sweden	Europe	Eriksson K, Nise L, Kats A, Luttropp E, Catrina AI, Askling J, Jansson L, Alfredsson L, Klareskog L, Lundberg K, Yucel-Lindberg T. Prevalence of Periodontitis in Patients with Established Rheumatoid Arthritis: A Swedish Population Based Case-Control Study. PLoS One. 2016 May 20;11(5):e0155956.
83	Ceccarelli et al., 2019	Periodontitis and rheumatoid arthritis: The same inflammatory mediators?	Mediators of Inflammation	English	9	Sapienza University of Rome	Italy	Europe	Ceccarelli F, Saccucci M, Di Carlo G, Lucchetti R, Pilloni A, Pranno N, Luzzi V, Valesini G, Polimeni A. Periodontitis and Rheumatoid Arthritis: The Same Inflammatory Mediators? Mediators Inflamm. 2019 May 5;2019:6034546.
84	Savioli et al., 2012	Persistent periodontal disease hampers anti-tumor necrosis factor treatment response in	Journal of Clinical Rheumatology	English	9	University of Sao Paulo	Brazil	America	Savioli C, Ribeiro AC, Fabri GM, Calich AL, Carvalho J, Silva CA, Viana VS, Bonfá E, Siqueira JT. Persistent periodontal disease hampers anti-tumor necrosis factor treatment response in rheumatoid arthritis. J Clin Rheumatol. 2012 Jun;18(4):180-4.

		rheumatoid arthritis							
85	Bello-Gualtero et al., 2016	Periodontal disease in individuals with a genetic risk of developing arthritis and early rheumatoid arthritis: A cross-sectional study	Journal of Periodontology	English	10	Universidad El Bosque	Colombia	America	Bello-Gualtero JM, Lafaurie GI, Hoyos LX, Castillo DM, De-Avila J, Munevar JC, Unriza S, Londoño J, Valle-Oñate R, Romero-Sánchez C. Periodontal Disease in Individuals with a Genetic Risk of Developing Arthritis and Early Rheumatoid Arthritis: A Cross-Sectional Study. J Periodontol. 2016 Apr;87(4):346-56.
86	Scher, Bretz and Abramson, 2014	Periodontal disease and subgingival microbiota as contributors for rheumatoid arthritis pathogenesis: Modifiable risk factors?	Current Opinion in Rheumatology	English	3	New York University Hospital for Joint Diseases	United States	America	Scher JU, Bretz WA, Abramson SB. Periodontal disease and subgingival microbiota as contributors for rheumatoid arthritis pathogenesis: modifiable risk factors? Curr Opin Rheumatol. 2014 Jul;26(4):424-9.
87	Äyräväinen et al., 2017	Periodontitis in early and chronic rheumatoid arthritis: A prospective follow-up study in Finnish population	BMJ Open	English	7	University of Helsink	Finland	Europe	Äyräväinen L, Leirisalo-Repo M, Kuuliala A, Ahola K, Koivuniemi R, Meurman JH, Heikkinen AM. Periodontitis in early and chronic rheumatoid arthritis: a prospective follow-up study in Finnish population. BMJ Open. 2017 Jan 31;7(1):e011916.

88	Potikuri et al., 2012	Periodontal disease is significantly higher in non-smoking treatment-naive rheumatoid arthritis patients: Results from a case-control study	Annals of the Rheumatic Diseases	English	8	Nizam's Institute of Medical Sciences	India	Asia	Potikuri D, Dannana KC, Kanchinadam S, Agrawal S, Kancharla A, Rajasekhar L, Pothuraju S, Gumdal N. Periodontal disease is significantly higher in non-smoking treatment-naive rheumatoid arthritis patients: results from a case-control study. Ann Rheum Dis. 2012 Sep;71(9):1541-4.
89	Kobayashi et al., 2014	Assessment of interleukin-6 receptor inhibition therapy on periodontal condition in patients with rheumatoid arthritis and chronic periodontitis	Journal of Periodontology	English	9	Niigata University	Japan	Asia	Kobayashi T, Okada M, Ito S, Kobayashi D, Ishida K, Kojima A, Narita I, Murasawa A, Yoshie H. Assessment of interleukin-6 receptor inhibition therapy on periodontal condition in patients with rheumatoid arthritis and chronic periodontitis. J Periodontol. 2014 Jan;85(1):57-67.
90	Trombone et al., 2010	Periodontitis and arthritis interaction in mice involves a shared hyper-inflammatory genotype and functional	Genes and Immunity	English	10	University of Sao Paulo	Brazil	America	Trombone AP, Claudino M, Colavite P, de Assis GF, Avila-Campos MJ, Silva JS, Campanelli AP, Ibañez OM, De Franco M, Garlet GP. Periodontitis and arthritis interaction in mice involves a shared hyper-inflammatory genotype and functional immunological interferences. Genes Immun. 2010 Sep;11(6):479-89.

		immunological interferences							
91	Kobayashi et al., 2014	Periodontal and serum protein profiles in patients with rheumatoid arthritis treated with tumor necrosis factor inhibitor adalimumab	Journal of Periodontology	English	11	Niigata University	Japan	Asia	Kobayashi T, Yokoyama T, Ito S, Kobayashi D, Yamagata A, Okada M, Oofusa K, Narita I, Murasawa A, Nakazono K, Yoshie H. Periodontal and serum protein profiles in patients with rheumatoid arthritis treated with tumor necrosis factor inhibitor adalimumab. J Periodontol. 2014 Nov;85(11):1480-8.
92	Esen et al., 2012	The effects of chronic periodontitis and rheumatoid arthritis on serum and gingival crevicular fluid total antioxidant/oxidant status and oxidative stress index	Journal of Periodontology	English	6	Erciyes University	Turkey	Asia	Esen C, Alkan BA, Kırnap M, Akgül O, Işıkoğlu S, Erel O. The effects of chronic periodontitis and rheumatoid arthritis on serum and gingival crevicular fluid total antioxidant/oxidant status and oxidative stress index. J Periodontol. 2012 Jun;83(6):773-9.
93	Hao et al., 2015	Deficiency of cathepsin K prevents inflammation and bone erosion in rheumatoid	FEBS Letters	English	7	University of Alabama	United States	America	Hao L, Zhu G, Lu Y, Wang M, Jules J, Zhou X, Chen W. Deficiency of cathepsin K prevents inflammation and bone erosion in rheumatoid arthritis and periodontitis and reveals its shared osteoimmune role. FEBS Lett. 2015 May 22;589(12):1331-1339.

		arthritis and periodontitis and reveals its shared osteoimmune role							
94	Bozkurt et al., 2006	Anti-inflammatory cytokines in gingival crevicular fluid in patients with periodontitis and rheumatoid arthritis: A preliminary report	Cytokine	English	5	Süleyman Demirel University	Turkey	Asia	Bozkurt FY, Yetkin Ay Z, Berker E, Tepe E, Akkuş S. Anti-inflammatory cytokines in gingival crevicular fluid in patients with periodontitis and rheumatoid arthritis: a preliminary report. Cytokine. 2006 Aug;35(3-4):180-5.
95	Courbon et al., 2019	Porphyromonas gingivalis experimentally induces periodontis and an anti-CCP2-associated arthritis in the rat	Annals of the Rheumatic Diseases	English	9	University Hospital of Saint-Etienne	France	Europe	Courbon G, Rinaudo-Gaujous M, Blasco-Baque V, Auger I, Caire R, Mijola L, Vico L, Paul S, Marotte H. Porphyromonas gingivalis experimentally induces periodontis and an anti-CCP2-associated arthritis in the rat. Ann Rheum Dis. 2019 May;78(5):594-599.
96	Ramamurthy et al., 2005	Experimental arthritis in rats induces Biomarkers of periodontitis which are ameliorated by gene therapy with	Journal of Periodontology	English	4	State University of New York	United States	America	Ramamurthy NS, Greenwald RA, Celiker MY, Shi EY. Experimental arthritis in rats induces Biomarkers of periodontitis which are ameliorated by gene therapy with tissue inhibitor of matrix metalloproteinases. J Periodontol. 2005 Feb;76(2):229-33.

		tissue inhibitor of matrix metalloproteinases							
97	Cheng et al., 2021	Dysbiosis in the oral microbiomes of anti-CCP positive individuals at risk of developing rheumatoid arthritis	Annals of the Rheumatic Diseases	English	10	University of Leeds	United Kingdom	Europe	Cheng Z, Do T, Mankia K, Meade J, Hunt L, Clerehugh V, Speirs A, Tugnait A, Emery P, Devine D. Dysbiosis in the oral microbiomes of anti-CCP positive individuals at risk of developing rheumatoid arthritis. Ann Rheum Dis. 2021 Feb;80(2):162-168.
98	Chou et al., 2015	Rheumatoid arthritis risk associated with periodontitis exposure: A nationwide, population-based cohort study	Plos One	English	5	Taichung Veterans General Hospital	Taiwan	Asia	Chou YY, Lai KL, Chen DY, Lin CH, Chen HH. Rheumatoid Arthritis Risk Associated with Periodontitis Exposure: A Nationwide, Population-Based Cohort Study. PLoS One. 2015 Oct 1;10(10):e0139693.
99	Bıyıkoğlu et al., 2013	Periodontal therapy in chronic periodontitis lowers gingival crevicular fluid interleukin-1beta and DAS28 in rheumatoid arthritis patients	Rheumatology International	English	7	Ege University	Turkey	Asia	Bıyıkoğlu B, Buduneli N, Aksu K, Nalbantsoy A, Lappin DF, Evrenosoğlu E, Kinane DF. Periodontal therapy in chronic periodontitis lowers gingival crevicular fluid interleukin-1beta and DAS28 in rheumatoid arthritis patients. Rheumatol Int. 2013 Oct;33(10):2607-16.

100	Cosgarea et al., 2019	Effects of non-surgical periodontal therapy on periodontal laboratory and clinical data as well as on disease activity in patients with rheumatoid arthritis	Clinical Oral Investigations	English	9	Philipps University Marburg	Germany	Europe	Cosgarea R, Tristiu R, Dumitru RB, Arweiler NB, Rednic S, Sirbu CI, Lascu L, Sculean A, Eick S. Effects of non-surgical periodontal therapy on periodontal laboratory and clinical data as well as on disease activity in patients with rheumatoid arthritis. Clin Oral Investig. 2019 Jan;23(1):141-151.
