# Supplementary material for: Mapping scientific knowledge on the association between periodontal disease and rheumatoid arthritis: A bibliometric and altmetric analysis of the 100 most-cited articles
Source: Med Oral Patol Oral Cir Bucal. 2026 Apr 9;31(5):e685–96. doi: 10.4317/medoral.28134 (PMC13636455; doi:10.4317/medoral.28134)
Supplement: Supplementary file 2 [file medoral-31-05-e685-s002.pdf]

Supllementary Table II. Indicators of design, theme, and impact of the 100 selected articles on the relationship between Periodontal Disease and Rheumatoid Arthritis.

Ranking	Citation, year	Study design	Study topic	International collaboration	Citations (Scopus)	Citations (Web of Science)	Altmetric score
1	Wegner et al., 2010	Laboratory	Molecular mechanisms and biomarkers	Yes	577	494	12
2	Konig et al., 2016	Laboratory	Molecular mechanisms and biomarkers	Yes	462	418	463
3	Scher et al., 2012	Observational	Molecular mechanisms and biomarkers	Yes	403	363	18
4	De Pablo et al., 2009	Review	Reviews and general concepts	No	396	361	20
5	De Pablo, Dietrich and McAlindon, 2008	Observational	Epidemiology and clinical association	No	353	303	0
6	Potempa, Mydel and Koziel, 2017	Review	Reviews and general concepts	Yes	352	322	0
7	Mercado et al., 2001	Review	Epidemiology and clinical association	No	341	281	19
8	Mikuls et al., 2014	Observational	Molecular mechanisms and biomarkers	Yes	339	294	10
9	Rosenstein et al., 2004	Review	Molecular mechanisms and biomarkers	No	314	268	6
10	Pischon et al., 2008	Observational	Epidemiology and clinical association	No	311	268	28
11	Lundberg et al., 2010	Review	Molecular mechanisms and biomarkers	Yes	295	272	0
12	Ortiz et al., 2009	Clinical trial	Treatment and therapeutic impact	Yes	272	228	42
13	Mercado et al., 2000	Observational	Epidemiology and clinical association	No	264	216	15
14	Bartold, Marshall and Haynes, 2005	Review	Reviews and general concepts	No	241	209	0
15	Mikuls et al., 2009	Laboratory	Molecular mechanisms and biomarkers	No	231	197	0
16	Dissick et al., 2010	Clinical trial	Epidemiology and clinical association	No	215	184	1

17	Maresz et al., 2013	Laboratory	Experimental models and animal studies	Yes	214	198	130
18	Kaur, White and Bartold, 2013	Review	Epidemiology and clinical association	No	210	189	51
19	Mikuls et al., 2012	Observational	Molecular mechanisms and biomarkers	No	200	171	3
20	Hitchon et al., 2010	Observational	Molecular mechanisms and biomarkers	Yes	193	167	0
21	Martinez-Martinez et al., 2009	Observational	Molecular mechanisms and biomarkers	Yes	191	166	8
22	Detert et al., 2010	Review	Epidemiology and clinical association	No	187	176	21
23	Al-Katma et al., 2007	Clinical trial	Treatment and therapeutic impact	No	187	154	0
24	De Smit et al., 2012	Observational	Molecular mechanisms and biomarkers	No	183	157	3
25	Koziel, Mydel and Potempa, 2014	Review	Reviews and general concepts	Yes	179	165	6
26	Fuggle et al., 2016	Review	Epidemiology and clinical association	No	178	163	0
27	Quirke et al., 2014	Laboratory	Molecular mechanisms and biomarkers	Yes	178	158	2
28	Mercado, Marshall and Bartold, 2003	Review	Epidemiology and clinical association	No	178	151	17
29	Sato et al., 2017	Laboratory	Experimental models and animal studies	No	176	161	27
30	Aquino et al., 2014	Laboratory	Experimental models and animal studies	Yes	174	156	0
31	Kässer et al., 1997	Observational	Treatment and therapeutic impact	No	172	139	3
32	De Molon et al., 2019	Review	Molecular mechanisms and biomarkers	Yes	168	153	0
33	Chen et al., 2013	Observational	Reviews and general concepts	No	160	150	1
34	Mayer, Balbir-Gurman and Machtei, 2009	Observational	Treatment and therapeutic impact	No	150	153	0
35	Marotte et al., 2006	Observational	Epidemiology and clinical association	No	146	122	0

36	Bingham and Moni, 2013	Review	Molecular mechanisms and biomarkers	No	141	122	0
37	Ribeiro, Leão and Novaes, 2005	Observational	Epidemiology and clinical association	No	135	110	0
38	Kharlamova et al., 2016	Observational	Molecular mechanisms and biomarkers	Yes	134	121	4
39	Harvey et al., 2013	Laboratory	Molecular mechanisms and biomarkers	No	132	116	1
40	Nesse et al., 2012	Observational	Molecular mechanisms and biomarkers	Yes	131	115	1
41	Persson, 2012	Review	Molecular mechanisms and biomarkers	No	124	80	1
42	Pers et al., 2008	Observational	Treatment and therapeutic impact	No	122	112	0
43	Araújo, Melo and Lima, 2015	Review	Epidemiology and clinical association	No	120	82	27
44	Demmer et al., 2011	Observational	Epidemiology and clinical association	Yes	119	107	4
45	Rodríguez-Lozano et al., 2019	Observational	Epidemiology and clinical association	No	118	107	25
46	Kaur et al., 2014	Review	Molecular mechanisms and biomarkers	No	116	86	8
47	Ogrendik et al., 2005	Laboratory	Molecular mechanisms and biomarkers	Yes	115	89	0
48	Ishida et al., 2012	Observational	Molecular mechanisms and biomarkers	No	110	100	3
49	Lappin et al., 2013	Observational	Molecular mechanisms and biomarkers	Yes	109	100	7
50	Berthelot and Le Goff, 2010	Review	Reviews and general concepts	No	109	89	3
51	Kassem et al., 2015	Laboratory	Experimental models and animal studies	Yes	106	103	4
52	Joseph et al., 2013	Observational	Epidemiology and clinical association	No	104	97	0
53	Cheng et al., 2017	Review	Epidemiology and clinical association	No	104	93	4
54	Cantley et al., 2011	Laboratory	Epidemiology and clinical association	No	103	95	5

55	Okada et al., 2013	Clinical trial	Treatment and therapeutic impact	No	103	84	0
56	Mirrieless et al., 2010	Observational	Molecular mechanisms and biomarkers	Yes	100	88	0
57	Marchesan et al., 2013	Laboratory	Experimental models and animal studies	Yes	99	92	0
58	Reichert et al., 2013	Observational	Molecular mechanisms and biomarkers	No	98	89	20
59	Corrêa et al., 2019	Observational	Epidemiology and clinical association	Yes	98	82	1
60	Gómez-Bañuelos et al., 2019	Review	Molecular mechanisms and biomarkers	No	96	88	0
61	Laugisch et al., 2016	Observational	Molecular mechanisms and biomarkers	Yes	94	90	2
62	Mankia et al., 2019	Observational	Epidemiology and clinical association	No	93	89	0
63	González-Febles and Sanz, 2021	Review	Treatment and therapeutic impact	No	93	86	1
64	Mangat et al., 2010	Review	Molecular mechanisms and biomarkers	Yes	92	77	3
65	Liao et al., 2009	Review	Molecular mechanisms and biomarkers	No	91	76	4
66	Arkema, Karlson and Costenbader, 2010	Observational	Epidemiology and clinical association	No	89	78	0
67	Konig et al., 2015	Laboratory	Molecular mechanisms and biomarkers	No	88	74	5
68	Kriebel et al., 2018	Review	Molecular mechanisms and biomarkers	Yes	88	74	0
69	Queiroz-Junior et al., 2011	Laboratory	Experimental models and animal studies	No	85	79	0
70	De Pablo et al., 2014	Observational	Molecular mechanisms and biomarkers	No	85	73	8
71	Leech and Bartold, 2015	Review	Reviews and general concepts	No	84	79	1
72	Erciyas et al., 2013	Observational	Epidemiology and clinical association	No	84	64	16
73	Lopez-Oliva et al., 2018	Clinical trial	Molecular mechanisms and biomarkers	Yes	82	77	42

74	Bartold and Lopez-Oliva, 2020	Review	Reviews and general concepts	Yes	82	72	1
75	Farquharson, Butcher and Culshaw, 2012	Review	Reviews and general concepts	No	82	61	1
76	Tolo and Jorkjend, 1990	Observational	Molecular mechanisms and biomarkers	No	81	76	3
77	Bartold et al., 2010	Laboratory	Experimental models and animal studies	No	80	69	0
78	Eriksson et al., 2019	Observational	Molecular mechanisms and biomarkers	No	79	69	0
79	Pinho et al., 2009	Clinical trial	Treatment and therapeutic impact	No	78	62	0
80	Payne et al., 2015	Review	Reviews and general concepts	No	78	Not found	1
81	Perricone et al., 2019	Review	Reviews and general concepts	Yes	77	69	0
82	Eriksson et al., 2016	Observational	Epidemiology and clinical association	No	75	70	13
83	Ceccarelli et al., 2019	Review	Molecular mechanisms and biomarkers	No	75	64	9
84	Savioli et al., 2012	Observational	Treatment and therapeutic impact	No	75	59	0
85	Bello-Gualtero et al., 2016	Observational	Molecular mechanisms and biomarkers	No	73	71	3
86	Scher, Bretz and Abramson, 2014	Review	Molecular mechanisms and biomarkers	No	71	64	0
87	Äyräväinen et al., 2017	Observational	Epidemiology and clinical association	No	71	64	0
88	Potikuri et al., 2012	Observational	Epidemiology and clinical association	No	70	65	8
89	Kobayashi et al., 2014	Clinical trial	Molecular mechanisms and biomarkers	No	70	62	0
90	Trombone et al., 2010	Laboratory	Experimental models and animal studies	No	70	59	0
91	Kobayashi et al., 2014	Observational	Molecular mechanisms and biomarkers	No	70	56	0
92	Esen et al., 2012	Observational	Molecular mechanisms and biomarkers	No	69	67	0

93	Hao et al., 2015	Laboratory	Molecular mechanisms and biomarkers	Yes	69	67	0
94	Bozkurt et al., 2006	Observational	Molecular mechanisms and biomarkers	No	69	65	0
95	Courbon et al., 2019	Laboratory	Experimental models and animal studies	No	69	61	0
96	Ramamurthy et al., 2005	Laboratory	Experimental models and animal studies	No	68	60	0
97	Cheng et al., 2021	Observational	Molecular mechanisms and biomarkers	Yes	67	63	0
98	Chou et al., 2015	Observational	Epidemiology and clinical association	No	67	58	9
99	Bıyıkoğlu et al., 2013	Clinical trial	Treatment and therapeutic impact	Yes	67	55	0
100	Cosgarea et al., 2019	Clinical trial	Treatment and therapeutic impact	Yes	67	54	3
