# Supplementary material for: Mapping scientific knowledge on the association between periodontal disease and rheumatoid arthritis: A bibliometric and altmetric analysis of the 100 most-cited articles
Source: Med Oral Patol Oral Cir Bucal. 2026 Apr 9;31(5):e685–96. doi: 10.4317/medoral.28134 (PMC13636455; doi:10.4317/medoral.28134)
Supplement: Supplementary file 3 [file medoral-31-05-e685-s003.pdf]

Supllementary Table III. Comparison of citations received in the Scopus and WoS databases according to decade of publication, continent, international collaboration, study design, and study topic.

Variables	Mean (Scopus)	SD	*P*-value	Mean (WoS)	SD	*P*-value
**Decade**						
1990s	126.50	64.347	**0.016 †***	107.50	44.548	**0.037†***
2000s	199.05	102.284		171.15	88.975	
2010s	137.03	95.172		119.91	85.651	
2020s	80.67	13.051		73.67	11.590	
**Continent**						
America	168.06	110.842	**0.043†***	144.38	100.834	0.065†
Asia	98.44	35.892		87,63	36.098	
Europe	144.44	101.371		127.64	89.314	
Oceania	166.45	87.597		142.09	71.969	
**International collaboration**						
Yes	175.55	126.381	0.143‡	156.03	111.573	0.059 ‡
No	133.73	77.794		114.97	68.725	
**Study design**						
Clinical trial	126.78	77.314	0.469†	106.67	64.971	0.568†
Laboratory	162.95	136.766		144.53	119.343	
Observational	137.00	83.957		120.81	72.441	
Review	158.73	94.479		135.73	88.832	
**Study topic**						
Epidemiology and clinical association	153.32	85.597	0.586†	132.56	72.160	0.631†
Experimental models and animal studies	144.10	53.548		103.80	50.104	
Molecular mechanisms and biomarkers	152.40	114.521		133.42	100.610	
Reviews and general concepts	167.27	115.351		143.36	113.646	
Treatment and therapeutic impact	126.00	64.211		107.82	55.515	

Legend: SD - standard deviation, WoS – Web of Science, § Mann-Whitney test, † Kruskal-Wallis test, *Statistical association ($p<0.05$).
