# Supplementary material for: Mapping scientific knowledge on the association between periodontal disease and rheumatoid arthritis: A bibliometric and altmetric analysis of the 100 most-cited articles
Source: Med Oral Patol Oral Cir Bucal. 2026 Apr 9;31(5):e685–96. doi: 10.4317/medoral.28134 (PMC13636455; doi:10.4317/medoral.28134)

**Supplementary Figure 1.** Trends in search interest for "periodontal disease" and "rheumatoid arthritis" over time and across regions (Google Trends). The numbers indicate the relative research interest during the analyzed period: a value of 100 represents the peak popularity of the term, 50 corresponds to half of that popularity, and 0 indicates insufficient data.

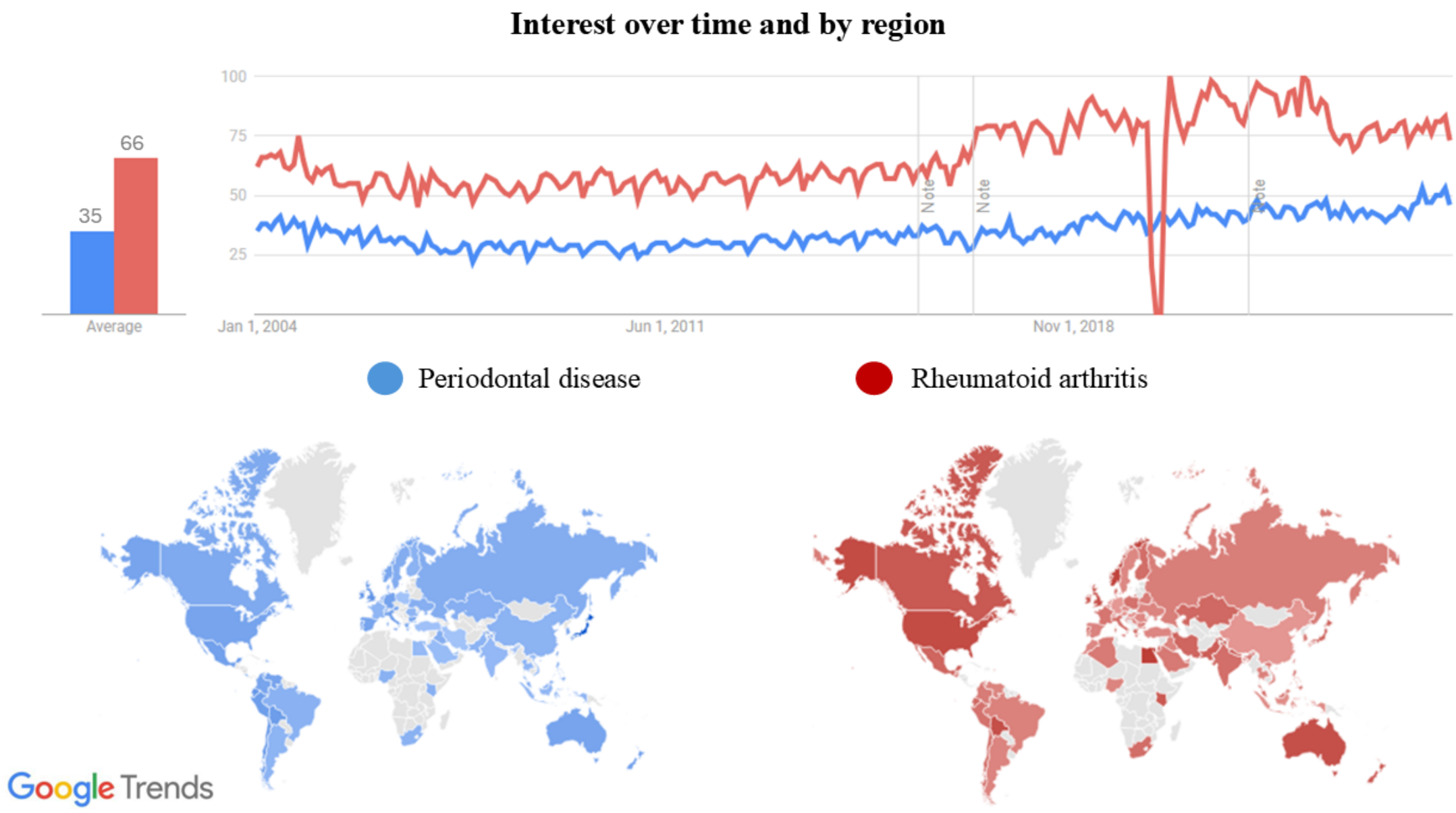

Supplement: Supplementary file 4 [file medoral-31-05-e685-s004.pdf]
